# An inorganic liquid crystalline dispersion with 2D ferroelectric moieties

**DOI:** 10.1093/nsr/nwae108

**Published:** 2024-03-21

**Authors:** Ziyang Huang, Zehao Zhang, Rongjie Zhang, Baofu Ding, Liu Yang, Keyou Wu, Youan Xu, Gaokuo Zhong, Chuanlai Ren, Jiarong Liu, Yugan Hao, Menghao Wu, Teng Ma, Bilu Liu

**Affiliations:** Shenzhen Graphene Centre, Shenzhen Key Laboratory of Advanced Layered Materials for Value-added Applications, Tsinghua−Berkeley Shenzhen Institute and Institute of Materials Research, Tsinghua Shenzhen International Graduate School, Tsinghua University, Shenzhen 518055, China; Shenzhen Graphene Centre, Shenzhen Key Laboratory of Advanced Layered Materials for Value-added Applications, Tsinghua−Berkeley Shenzhen Institute and Institute of Materials Research, Tsinghua Shenzhen International Graduate School, Tsinghua University, Shenzhen 518055, China; Shenzhen Graphene Centre, Shenzhen Key Laboratory of Advanced Layered Materials for Value-added Applications, Tsinghua−Berkeley Shenzhen Institute and Institute of Materials Research, Tsinghua Shenzhen International Graduate School, Tsinghua University, Shenzhen 518055, China; Shenzhen Graphene Centre, Shenzhen Key Laboratory of Advanced Layered Materials for Value-added Applications, Tsinghua−Berkeley Shenzhen Institute and Institute of Materials Research, Tsinghua Shenzhen International Graduate School, Tsinghua University, Shenzhen 518055, China; Institute of Technology for Carbon Neutrality, Faculty of Materials Science and Engineering, Shenzhen Institute of Advanced Technology, Chinese Academy of Sciences, Shenzhen 518055, China; School of Physics and Institute for Quantum Science and Engineering, School of Chemistry and Institute of Theoretical Chemistry, Huazhong University of Science and Technology, Wuhan 430074, China; Shenzhen Graphene Centre, Shenzhen Key Laboratory of Advanced Layered Materials for Value-added Applications, Tsinghua−Berkeley Shenzhen Institute and Institute of Materials Research, Tsinghua Shenzhen International Graduate School, Tsinghua University, Shenzhen 518055, China; Shenzhen Graphene Centre, Shenzhen Key Laboratory of Advanced Layered Materials for Value-added Applications, Tsinghua−Berkeley Shenzhen Institute and Institute of Materials Research, Tsinghua Shenzhen International Graduate School, Tsinghua University, Shenzhen 518055, China; Xi'an Research Institute of High Technology, Xi'an 710025, China; Shenzhen Institute of Advanced Technology, Chinese Academy of Sciences, Shenzhen 518055, China; Shenzhen Institute of Advanced Technology, Chinese Academy of Sciences, Shenzhen 518055, China; Shenzhen Graphene Centre, Shenzhen Key Laboratory of Advanced Layered Materials for Value-added Applications, Tsinghua−Berkeley Shenzhen Institute and Institute of Materials Research, Tsinghua Shenzhen International Graduate School, Tsinghua University, Shenzhen 518055, China; Shenzhen Graphene Centre, Shenzhen Key Laboratory of Advanced Layered Materials for Value-added Applications, Tsinghua−Berkeley Shenzhen Institute and Institute of Materials Research, Tsinghua Shenzhen International Graduate School, Tsinghua University, Shenzhen 518055, China; School of Physics and Institute for Quantum Science and Engineering, School of Chemistry and Institute of Theoretical Chemistry, Huazhong University of Science and Technology, Wuhan 430074, China; Department of Applied Physics, Hong Kong Polytechnic University, Hong Kong, China; Shenzhen Graphene Centre, Shenzhen Key Laboratory of Advanced Layered Materials for Value-added Applications, Tsinghua−Berkeley Shenzhen Institute and Institute of Materials Research, Tsinghua Shenzhen International Graduate School, Tsinghua University, Shenzhen 518055, China

**Keywords:** 2D materials, layered minerals, vermiculite, ferroelectric, inorganic liquid crystal, Kerr coefficient

## Abstract

Electro-optical effect-based liquid crystal devices have been extensively used in optical modulation techniques, in which the Kerr coefficient reflects the sensitivity of the liquid crystals and determines the strength of the device’s operational electric field. The Peterlin–Stuart theory and the O'Konski model jointly indicate that a giant Kerr coefficient could be obtained in a material with both a large geometrical anisotropy and an intrinsic polarization, but such a material is not yet reported. Here we reveal a ferroelectric effect in a monolayer two-dimensional mineral vermiculite. A large geometrical anisotropy factor and a large inherent electric dipole together raise the record value of Kerr coefficient by an order of magnitude, till 3.0 × 10^−4^ m V^−2^. This finding enables an ultra-low operational electric field of 10^2^–10^4^ V m^−1^ and the fabrication of electro-optical devices with an inch-level electrode separation, which has not previously been practical. Because of its high ultraviolet stability (decay <1% under ultraviolet exposure for 1000 hours), large-scale production, and energy efficiency, prototypical displayable billboards have been fabricated for outdoor interactive scenes. This work provides new insights for both liquid crystal optics and two-dimensional ferroelectrics.

## Teaser

A century-old clay material is discovered to show ferroelectricity in its monolayer thickness limit, leading to a record high electro-optical Kerr effect and sensitive electric field responsitivity.

## INTRODUCTION

Liquid crystal (LC) devices based on electro-optical effects have achieved huge success in the information era, where the electric field alters the alignment of anisotropic LC molecules, giving rise to a change in their observed optical properties and achieving light modulation [1–4]. The electro-optical Kerr effect, first reported in 1875 by John Kerr, is one typical representative of electro-optical effects [5,6]. Along with the research of blue phase LCs [7–10], Kerr effect devices that can avoid the use of alignment layers and transparent electrodes have attracted researchers' attention [11,12], making them competitive in many emerging fields, such as Kerr display and terahertz modulator [11–14]. In this effect, the Kerr coefficient that describes a quadratic relationship between induced birefringence and electric field strength is a key parameter, which reflects not only the strength of the electro-birefringence effect, but also the sensitivity of the alignment of LC molecules in response to electric stimulus [9,10]. However, values of the Kerr coefficients are usually within the range of 10^−11^–10^−7^ m V^−2^ for commercial LCs, and all require an operational electric field of >10^6^ V m^−1^ for LC electro-optical devices [9,10]. Therefore, it is an essential topic to improve the intrinsic electro-optical sensitivity of LCs and decrease the operational electric field by developing new LC materials and revealing their intriguing properties, where the study of the Kerr coefficient is an appropriate platform.

To obtain a large Kerr coefficient, the Peterlin–Stuart theory and the O'Konski model have been studied since the 1940s, and these scientists have proposed that materials with an intrinsic polarization and a large geometrical anisotropy are promising [15–17]. Organic ferroelectric LCs and inorganic LCs formed from low-dimensional materials have been developed to meet these criteria [18–24]. Organic ferroelectric LCs with a macroscopic polarization order have a more sensitive response than their traditional counterparts [25,26], but their molecular scale with a small geometrical anisotropy limits further improvement of the Kerr coefficient. Two-dimensional (2D) materials with a micrometer-scale length and an atomic level thickness are believed to have the largest geometrical anisotropy among all the other materials in dispersion [27]. As the preparation techniques of 2D materials become fully developed, increase of the Kerr coefficient is especially challenging, because the geometrical anisotropy approaches the upper limit [28]. In this regard, ideal Kerr media, namely inorganic LCs or LC-like systems based on 2D materials with both an intrinsic polarization and a large geometrical anisotropy, are urgently needed, while such LCs have not been reported so far.

Here we show such a 2D vermiculite (VMT) liquid crystalline dispersion with an anomalously large Kerr coefficient of 3.0 × 10^−4^ m V^−2^, which is an order of magnitude higher than all known media. The exfoliated 2D VMT has both a large geometrical anisotropy factor over 1500 and an intrinsic ferroelectricity, and these properties jointly contribute to the giant Kerr coefficient. For 2D VMT liquid crystalline dispersion, we establish a relationship among Kerr coefficient$\ K$, intrinsic polarization$\ P$, and geometrical anisotropy factor ${\mathrm{\gamma }}$, namely, $K \propto {{P}^2}{{{\mathrm{\gamma }}}^4}$, which has been experimentally verified. Thanks to the giant Kerr coefficient, it is now possible for us to demonstrate a large-area prototypical display with low-energy consumption for potential outdoor use.

## RESULTS AND DISCUSSION

**Figure 1.**
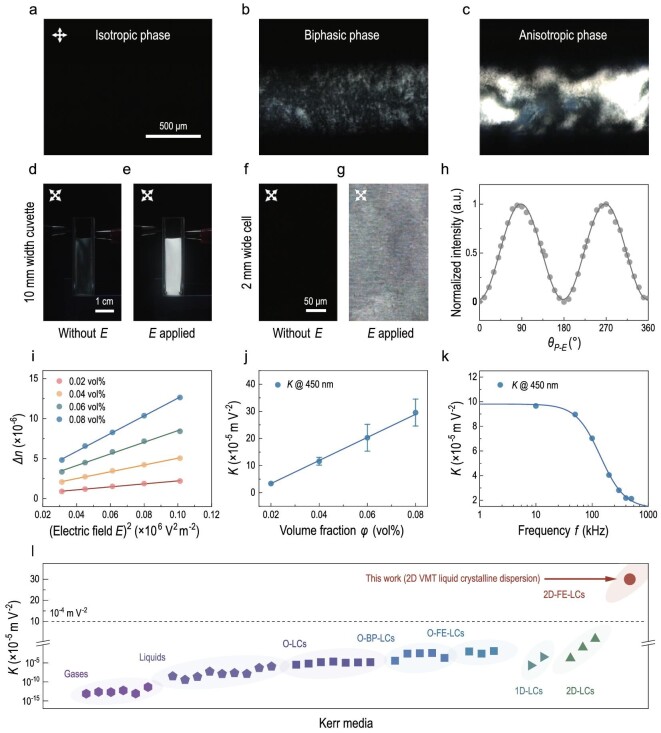
2D VMT liquid crystalline dispersion and its anomalously large Kerr coefficient. (a–c) Polarized optical microscopy images of 2D VMT liquid crystalline dispersion with volume fractions of (a) 0.08, (b) 0.25, and (c) 0.60 vol%. (d and e) Optical images of a cuvette filled with 2D VMT liquid crystalline dispersion with a volume fraction of 0.08 vol%. The cuvette is placed in front of a white backlight and in between a pair of crossed polarizers with (d) no electric field and (e) a transverse electric field of 1.0 × 10^3^ V m^−1^ (*E* perpendicular to the direction of optical path). (f and g) Polarized optical microscopy images of an LC cell containing 2D VMT liquid crystalline dispersion with (f) no electric field and (g) a transverse electric field of 1.0 × 10^4^ V m^−1^. (h) Normalized intensity of the transmitted light with ${{\theta }_{P - E}}$, the angle between the normal of the VMT platelet and the transmission axes of the polarizer. The intensity of the transmitted light with the polarization of incident light perpendicular to the electric field is higher than that parallel to the electric field. (i) Birefringence $\Delta n$ of 2D VMT liquid crystalline dispersion with volume fractions of 0.02, 0.04, 0.06 and 0.08 vol%. $\Delta n$ varies linearly with the square of the electric field *E* in fields below 3.2 × 10^3^ V m^−1^. (j) The Kerr coefficient *K* for 2D VMT liquid crystalline dispersions with volume fractions of 0.02, 0.04, 0.06 and 0.08 vol%. *K* for 2D VMT liquid crystalline dispersion with a volume fraction of 0.08 vol% is 3.0 × 10^−4^ m V^−2^. The frequency of all the electric fields used in (d–j) is 10 kHz. (k) Frequency dispersion of *K* in the range of 10 to 1000 kHz. Results in (h–k) are collected using a 450 nm laser as an incident light. (l) Comparison of the measured *K* values of 2D VMT liquid crystalline dispersion with those of gases, liquids, organic LCs and inorganic nanomaterial LCs reported in the literature, where O-LCs, O-BP-LCs, O-FE-LCs, 1D-LCs, 2D-LCs, and 2D-FE-LCs are classical organic LCs, organic blue phase LCs, organic ferroelectric LCs, inorganic LCs (or LC-like dispersion) based on one-dimensional materials, inorganic LCs (or LC-like dispersion) based on 2D materials, and inorganic LCs (or LC-like dispersion) based on 2D ferroelectric materials, respectively. The data we used are given in Table S2.

An aqueous 2D VMT dispersion was prepared by exfoliating bulk layered minerals. These bulk layered minerals are abundant in nature. Their nanoscale preparation, properties, and value-added uses have been intensively studied in these present decades [24,29–32]. For 2D VMT dispersion, a cation-exchange method was applied (see Methods and Fig. S1). During this process, the interlayer ions of VMT were replaced by cations in solution (e.g. Na^+^, Li^+^). The interlayer spacing of VMT expanded, so that the interlayer van der Waals force was weakened [24,31,32]. Hence, we were able to produce VMT monolayers and their dispersion (Fig. S2). Note that, the deionization treatment to decrease the ionic strength of the dispersion is a prerequisite in this study, because the ion-induced electrostatic screening and/or electrophoresis can lead to either a weakened impact of the electric field on the 2D VMT or large current leakage (Fig. S3). The 2D VMT dispersion had a greenish brown color and exhibited colloidal behavior, as evidenced by its Tyndall effect (Fig. S4a and b). A zeta potential of −48.1 mV indicated that the 2D VMT had a negatively charged surface (Fig. S4c), where the repulsive electrostatic force between adjacent platelets prevents their aggregation and consequently imparts stability to the dispersion. When placed under crossed polarizers, we observed that 2D VMT dispersion underwent a phase transition from an isotropic phase to biphasic and anisotropic ones with an increasing volume fraction at a static state (Fig. 1a–c). When shaken by hand, diluted 2D VMT dispersion exhibited flow-induced birefringent textures with a brush shape (Fig. S5a–c), where the texture was similar to other lyotropic liquid crystalline dispersions [20–22,33]. The dark brushes indicated that the dispersed 2D VMT platelets were temporarily parallel to one of the crossed polarizer axes, making the corresponding area to extinction. In contrast, the 2D VMT platelets collectively oriented in an intermediate direction in the bright regions. More fringes with birefringence colors (e.g. red, purple, and green) appeared as the volume fraction increased (Fig. S5d). These temporary ordered domains and birefringent textures disappeared both globally and locally within several minutes, and these dilute 2D VMT dispersions returned to an optically isotropic state after shaking (see Figs S6 and S7). Without a flow, a 2D VMT dispersion with a volume fraction of 0.40 vol% (or higher) showed a spontaneous Schlieren texture, corresponding to the anisotropic nematic phase (see both Fig. 1c and Fig. S5d). These results indicated that 2D VMT dispersion had liquid crystalline properties. Small angle X-ray scattering and rheological tests were also performed to observe the phase transition behavior. The elliptical small angle X-ray scattering pattern and the resultant profile showed that the 2D VMT dispersion with a volume fraction of 1.2 vol% became anisotropic, where the long axis of the elliptical pattern was coplanar with the normals of 2D VMT platelets (Fig. S8). In the rheological test, 2D VMT dispersion showed a volume-fraction–induced thickening effect and shear-induced thinning effect, which agreed with the lyotropic liquid crystalline nature of the 2D VMT dispersion (Fig. S9).

Figure 1d–g presents the electro-optical birefringence response of 2D VMT liquid crystalline dispersion. A cuvette filled with 2D VMT liquid crystalline dispersion appeared black in the absence of an electric field, because the disordered VMT platelets were optically isotropic, giving rise to optical extinction under crossed polarizers (Fig. 1d). It turned bright after applying a transverse electric field of 1.0 × 10^3^ V m^−1^ with a frequency of 10 kHz, due to the transmission of partial light from the backlight source (Fig. 1e), while an electric field of ∼10^6^ V m^−1^ is usually needed for conventional LCs. It indicates a drop of operational electric field strength by three orders of magnitude. This was also observed by polarized optical microscope (Fig. 1f and g). For an optical setup with birefringent medium sandwiched between two crossed polarizers, the transmitted intensity of light can be expressed as $I = {{I}_0}si{{n}^2}( {2{{\theta }_{LC - P}}} )si{{n}^2}( {\frac{\delta }{2}} )$, where *I* is the transmitted light intensity, ${{I}_0}$ is the light intensity after passing through the first polarizer, and ${{\theta }_{LC - P}}$ is the angle between the normal of the VMT platelet and the transmission axes of the polarizer [34–37]. The phase retardation $\delta $ is dependent on the optical path and induced birefringence $\Delta n$, where $\Delta n$ is controlled by the alignment of LC molecules. In this regard, the electric field induced alignment subsequently controls the light transmittance. In the absence of an electric stimulus, the 2D VMT liquid crystalline dispersion was initially optically isotropic due to the random orientation of 2D VMT platelets. When the platelets are aligned by external stimulus, an anisotropic absorption occurs (Fig. S10). The transmitted light intensity with the incident light polarized perpendicular to the electric field was higher than that parallel to the electric field (Fig. 1h). Besides, some textures that run parallel to the transverse electric field were also observed (Fig. S11). These results collectively indicate that the 2D VMT platelets aligned in parallel to the electric field *E*, i.e. their normals are perpendicular to *E*.

As *E* approaches 0, $\Delta n$ and *E* theoretically satisfy the relationship of $\Delta n = K\lambda {{E}^2}$ (*K* is the Kerr coefficient and $\lambda $ the incident wavelength), consistent with the experimental observation (Fig. 1i). Here, $\Delta n$ was determined by calculating the polarization parameters of azimuth angle and ellipticity (see Methods, Text S1, Table S1, Figs S12 and S13). For 2D VMT liquid crystalline dispersion with a volume fraction of 0.08 vol%, the Kerr coefficient was determined to be 3.0 × 10^−4^ m V^−2^ (Fig. 1j). It is worth noting that the Kerr coefficient of 2D VMT liquid crystalline dispersion is one order of magnitude higher than those of all known Kerr media, including organic blue phase LCs, organic ferroelectric LCs, and all inorganic nanomaterial LCs (see Fig. 1l and Table S2). Theoretically, the Peterlin–Stuart theory gives the basic relationship between the birefringence, optical anisotropy factor $\Delta g$ of a LC system and orientational order parameter [15,16]. Besides, the O'Konski model illustrates that $\Delta \alpha $, the anisotropy of excess electrical polarizabilities of the LC unit beyond the electrical polarizabilities of dispersant, and $\mu $, the inherent electric dipole of each LC unit, jointly determine the orientational order parameter [17]. Simply put, we showed that the Kerr coefficient was positively correlated with $\Delta g$, $\Delta \alpha $ and $\mu $ (Text S2). Measurements of saturated birefringence and the frequency dependence of the Kerr coefficient, experimentally gave that $\Delta g$, $\Delta \alpha $ and $| \mu |$ for 2D VMT liquid crystalline dispersion were −2.3 × 10^−12^ C^2^ J^−1^ m^−1^, −9.3 × 10^−27^ F m^2^ and 1.7 × 10^−23^ C m (5.1 × 10^6^ Debye), respectively (see Fig. 1k, Text S3, Text S4 and Fig. S14). The negative values of $\Delta g$ and $\Delta \alpha $ demonstrated an in-plane easy axis. Surprisingly, there existed an inherent electric dipole $| \mu |$ of 1.7 × 10^−23^ C m, which possibly indicated the ferroelectric nature of 2D VMT platelets.

**Figure 2.**
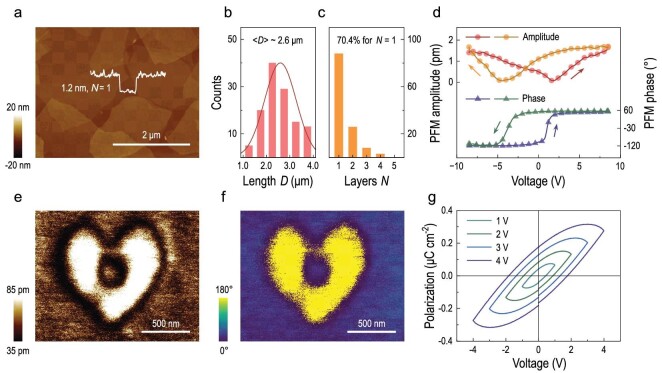
2D VMT and evidence of ferroelectricity. (a) Atomic force microscopy image of 2D VMT. The height profile shows a height difference of 1.2 nm between the substrate and the 2D VMT platelets. The platelets are assigned as VMT monolayers, i.e. $N = 1$, where *N* is the number of layers. (b and c) Statistics of (b) the length *D* and (c) the number of layers *N* of the 2D VMT. $\langle D \rangle $ is determined to be 2.6 μm and 70.4% of 2D VMT is monolayer. Bilayer ($N = 2$) and trilayer ($N = 3$) platelets account for 20.8% and 6.4%, respectively. (d) PFM amplitude and phase *versus* voltage hysteresis loops of 2D VMT. (e) PFM amplitude and (f) PFM phase maps of 2D VMT scanned after writing a heart pattern by applying a DC bias of −10 V and 10 V. (g) P–V hysteresis loops of a 2D VMT laminate film under different voltage biases at room temperature.

In order to fully understand the anomalously large Kerr coefficient, we first performed the morphology characterization on 2D VMT. Atomic force microscope and transmission electron microscope images revealed that 2D VMT had an average length $\langle D \rangle $ of 2.6 μm (see Fig. 2a and b, and Fig. S15a), and 70.4% of them had an average thickness of 1.3 nm, which were identified as VMT monolayers (see Fig. 2c and Fig. S15b). The proportions of bilayer and trilayer platelets were found to be 20.8% and 6.4%, respectively (Fig. S15b). The weighted average height $\langle H \rangle $ was thus determined to be 1.7 nm, giving a large geometrical anisotropy factor ${\mathrm{\gamma }}$ over 1500, where ${\mathrm{\gamma }} = \frac{{\langle D \rangle }}{{\langle H \rangle }}$ for 2D materials. By using the geometrical parameters above, we showed that the nematic phase transition of 2D VMT liquid crystalline dispersion theoretically occurs at 0.40 vol% (Text S5), which is close to that used in our experiment (see Fig. S5d). Therefore, the phase transition behavior also supported the results of morphology characterization.

To examine the inherent electric dipole, we attempted ferroelectric characterization using piezoresponse force microscopy (PFM), where 2D VMT was coated on an Au substrate (see Methods and Fig. S16). Upon applying an electric field, the amplitude and phase signal of the PFM, respectively, reflects the local change of morphology caused by the piezoelectric effect and polarization direction. PFM spectroscopy exhibited a well-defined butterfly-type loop in amplitude, and the phase loop had a hysteresis behavior with a polarization switching of 180° (Fig. 2d). DC poling voltages of −10 V and 10 V were then applied at different positions to write heart and box-in-box patterns on 2D VMT, where the induced polarization is conserved after removal of the DC bias for a ferroelectric material. The PFM images showed clear heart or box-in-box patterns in both amplitude and phase channel after poling, where the phase change of 180° was consistent with the result obtained from PFM spectroscopy (see Fig. 2e and f, and Fig. S17d and f). We note that both changes in morphology and background signal were negligible (Fig. S17a–c and e). A Kelvin probe force microscope also witnessed the lithographic pattern after poling with a surface potential change >100 mV (Fig. S18).

Macroscopic polarization versus an external electric field was also measured. A 40 μm-thick 2D VMT laminate film with VMT platelets parallel to each other was prepared by vacuum filtration (Fig. S19), and was used to ensure a large polarization signal. A series of macroscopic polarization versus voltage (P–V) loops were recorded at room temperature under different driven electric voltages at 0.1 kHz (Fig. 2g). It suggested the existence of electric dipoles in the 2D VMT laminate that can be switched by an external electric field. Note that the observed polarization may include a contribution from an unavoidable leakage current, leading to the unsaturated shapes of P–V loops. Such a feature is similar to other relaxor ferroelectric materials [38,39]. Since the contribution from leakage current can be largely suppressed at high frequencies [40,41], a series of P–V loops at high frequencies up to 100 kHz were measured. The switching and hysteresis behavior of P–V loops at high frequency are shown in Fig. S20, which gave a polarization of 0.03 μC cm^−2^ and confirmed the essential contribution from electric dipoles. Thus, we presumably ascribed the polarization to an inherent electric dipole. To reveal the origin of the inherent electric dipole, we performed first principles calculations on a monolayer VMT (see Methods). Text S6 and Fig. S21 show that the vacancies induced by elemental substitution (e.g. by Al and Fe) in a monolayer VMT is a possible origin of the inherent electric dipole and resultant ferroelectricity. Such a mechanism relies on the migration of protons from one side of the MgO_2_ sheet to the other. In this case, 2D VMT possessed a flipped polarization in both the in-plane and out-of-plane direction, and the in-plane polarization is larger than the out-of-plane polarization, which agreed with our experimental results (Fig. 1h, Figs S11 and S22).

Besides, it is worth noting that the lyotropic liquid crystalline dispersion with ferroelectric building units (i.e. 2D VMT platelets in this work) differs from the generally understood thermotropic ferroelectric LCs. One can observe the polar ordered domains and their transition controlled by a DC electric field in thermotropic ferroelectric LCs [19,42,43], while there was no similar phenomenon for 2D VMT liquid crystalline dispersion until DC electrophoresis took place (Fig. S23). In theory, interactions between molecules in ferroelectric LCs typically involve three fundamental mechanisms, i.e. charge-charge interaction ${{V}_{cc}}$, dipole-charge interaction ${{V}_{cd}}$, and dipole-dipole interaction ${{V}_{dd}}$. The strength of these interactions varies with the distance between adjacent molecules, respectively, following inverse relationships of the first, second, and third orders of the distance *r*, namely, ${{V}_{cc}} \propto {{r}^{ - 1}}$, ${{V}_{cd}} \propto {{r}^{ - 2}}$, and ${{V}_{dd}} \propto {{r}^{ - 3}}$. When molecules possess strong surface charges (Fig. S4c), charge-charge repulsion becomes the dominant force, pushing molecules away from each other to several hundreds of nanometers (726 nm for 2D VMT liquid crystalline dispersion, based on the Derjaguin–Landau–Verwey–Overbeek theory, see Text S7 and Fig. S24), thereby weakening both dipole-charge and dipole-dipole interaction to a negligible level. Thus, the individual ferroelectric nature of LC building units directly contributes to the increase of electro-optical sensitivity (i.e. the Kerr coefficient), rather than forming collective polar ordered domains as in the case of thermotropic ferroelectric LCs.

Nevertheless, these results are of importance when examining the giant Kerr coefficient of 2D VMT liquid crystalline dispersion. Text S8 and S9 give insights on the theoretical determination of optical anisotropy factor $\Delta g$ and excess electrical polarizability anisotropy $\Delta \alpha $ by using the geometrical parameters we obtained in Fig. 2a and b, and Fig. S15b (average length $\langle D \rangle $ of 2.6 μm, weight average height $\langle H \rangle $ of 1.7 nm, and a geometrical anisotropy factor ${\mathrm{\gamma }}$ of 1500). The $\Delta g$ and $\Delta \alpha $ were determined to be −2.2 × 10^−12^ C^2^ J^−1^ m^−1^ and −8.1 × 10^−27^ F m^2^, respectively, which agreed well with the experimental results (−2.3 × 10^−12^ C^2^ J^−1^ m^−1^ and −9.3 × 10^−27^ F m^2^). By combining these geometrical parameters with the polarization of 0.03 μC cm^−2^, we calculated the inherent electric dipole $| \mu |$ of 2D VMT liquid crystalline dispersion. Text S10 presents a $| \mu |$ of 1.8 × 10^−23^ C m, being close to 1.7 × 10^−23^ C m. Note that Text S11 shows that all the three aforementioned critical parameters ($| {\Delta g} |$, $| \mu |$ and $| {\Delta \alpha } |$) that determine the Kerr coefficient were monotonically increased with an increasing geometrical anisotropy factor of the LC units. For 2D VMT possessing an intrinsic ferroelectric response with a large geometrical anisotropy factor ${\mathrm{\gamma }}$ of **>**1500 and an intrinsic polarization *P* of 0.03 μC cm^−2^, the contribution of $| \mu |$, i.e. ${{( {\frac{\mu }{{{{k}_B}T}}} )}^2}$ of ∼1.9 × 10^−5^ m^2^ V^−2^ was much greater than that of $| {\Delta \alpha } |$, i.e. $\frac{{{\mathrm{\Delta }}\alpha }}{{{{k}_B}T}}$ of ∼2.3 × 10^−6^ m^2^ V^−2^ according to our calculation. In such a case, an approximate optimization gave a generalized formula of $K \propto {{P}^2}{{{\mathrm{\gamma }}}^4}$ that showed a strong positive correlation of Kerr coefficient with an intrinsic polarization *P* and a geometrical anisotropy factor ${\mathrm{\gamma }}$ (Text S11). To clearly explain this issue, we further verified the derived relationship experimentally. First, 2D VMT liquid crystalline dispersion with a volume fraction of 0.08 vol% was bath-sonicated for 30, 60 and 120 minutes. Figure S25 shows that the length of 2D VMT decreased with increasing durations of sonication, and their average lengths were determined to be 1.4, 1.0, and 0.7 μm. Since the as-exfoliated 2D VMT are monolayer dominant, a weight average height of 1.7 nm was taken into the calculation, which gave their average geometrical anisotropy factors of 841, 561 and 401. Figure S26 shows the results of Kerr experiments. The four liquid crystalline dispersions in which 2D VMT had average geometrical anisotropy factors of 1525 (without sonication), 841, 561 and 401 showed Kerr coefficients of 3.0 × 10^−4^, 3.4 × 10^−5^, 4.8 × 10^−6^, and 1.1 × 10^−6^ m V^−2^, respectively. Of note, the fitting curve gave a relationship of $K \propto {{{\mathrm{\gamma }}}^{4.2}}$, which is close to our derivation that $K \propto {{{\mathrm{\gamma }}}^4}$. Besides, the Kerr coefficients of 2D VMT liquid crystalline dispersion showed a negative quadratic dependence with temperature as $K \propto {{T}^{ - 2.2}}$ (Fig. S27). In principle, the relationship of $K \propto {{T}^{ - 2}}$ only occurred in the case where the contribution of $| \mu |$ dominates. Hence, we proposed that an extremely large geometrical anisotropy and an intrinsic polarization of 2D VMT jointly increased the sensitivity of the electro-optical Kerr effect to a new record of 3.0 × 10^−4^ m V^−2^, which is one order of magnitude higher than all known media.

**Figure 3.**
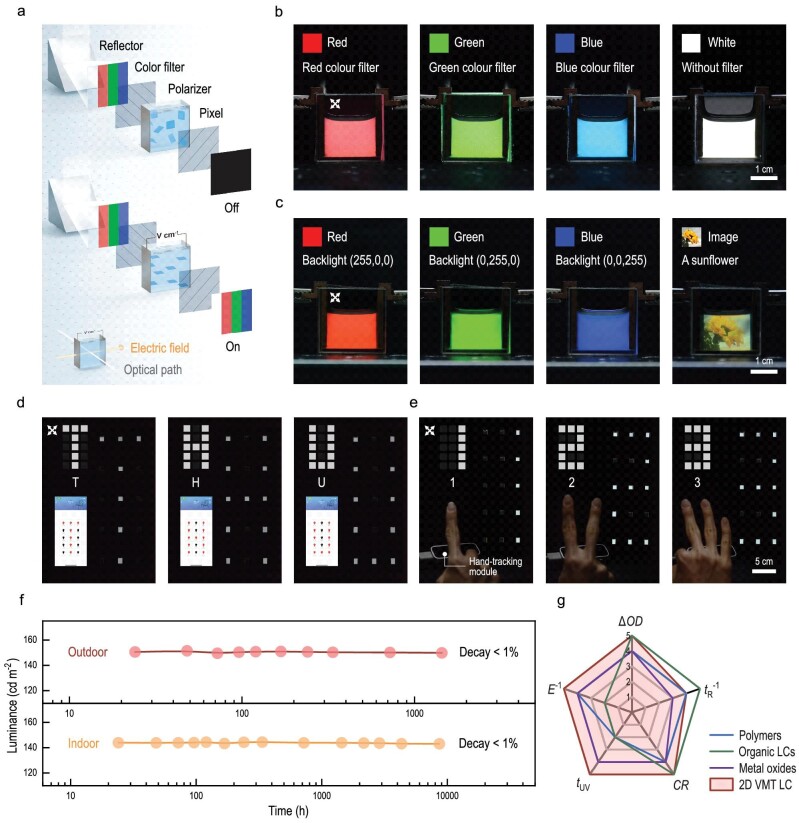
Proof-of-concept devices using 2D VMT liquid crystalline dispersion. (a) Schematic of a backlight-free displayable pixel using a reflector. The pixel appears black without an electric field. An electric field of 10^2^–10^4^ V m^−1^ is used to turn on the device. Color filters are used to get red, green and blue colors. The bottom left inset presents the device configuration with the electric field direction perpendicular to the optical path. (b) Optical images of a backlight-free displayable pixel showing red, green, blue, and white colors with an applied electric field of 4.0 × 10^3^ V m^−1^. (c) Optical images of a displayable pixel with a backlight module showing red, green, blue colors and a sunflower picture with an applied electric field of 4.0 × 10^3^ V m^−1^. (d) Pixel array remotely controlled by software. The array shows the letters ‘T’, ‘H’ and ‘U’ with an applied electric field of 8.0 × 10^3^ V m^−1^. (e) Pixel array remotely controlled by gestures of the controller. A hand-tracking module is equipped to capture the gesture. The array shows the numbers ‘1’, ‘2’ and ‘3’ with an applied electric field of 8.0 × 10^3^ V m^−1^. The frequency of all electric fields used is set as 10 kHz in (b–e). (f) Luminance of a 2D VMT liquid crystalline dispersion pixel during long-term outdoor and indoor stability tests. The decay is <1% in both cases. (g) Comprehensive comparison of a 2D VMT liquid crystalline dispersion device with those electrochromic techniques reported in the literature. Five parameters are used: outdoor stability (represented by life in ultraviolet irradiation, *t*_UV_), manipulating field (*E*^−1^), optical density (Δ*OD*), response time (*t*_R_^−1^), and contrast ratio (*CR*).

As a result, a giant Kerr coefficient makes it possible to fabricate a large-scale device with low energy consumption, where the operational electric field can be theoretically dropped from 10^6^ V m^−1^ for conventional LCs to 10^2^–10^4^ V m^−1^ for the 2D VMT liquid crystalline dispersion. Such a low operational field consequently helps overcome some potential operational problems like electrophoresis and electroplating, and enables the use of electrodes with macroscopic spacing in an electro-optical Kerr device. We designed and fabricated 2D VMT liquid crystalline dispersion based pixels with a size of 1.4 inch, which had two working modes with its backlight supplied from a light emitting diode screen for night use, or reflection of a solar beam for use during the day (Fig. 3a). The pixel was comprised of two electrodes separated in centimeter-scale space, supplying an electric field in the range of 10^2^–10^4^ V m^−1^. Since the direction of the electric field was perpendicular to the light path in the 2D VMT liquid crystalline dispersion pixel, transparent electrodes, such as indium tin oxide or fluorine doped tin oxide, were not required, permitting the use of cost-effective non-transparent metals, such as copper, as alternative electrodes. In the meantime, because of its wide optical band gap and corresponding absorption edge of >400 nm, 2D VMT liquid crystalline dispersion with a volume fraction of 0.04 vol% had an average transmittance of 88% for the visible light (Fig. S28). As a result, we loaded such dispersion into the pixel that can then be scaled up to inch level. With the use of color filters (Fig. 3b) or monochromic lights with different wavelengths (see Fig. Video S1), the pixel displayed red, green and blue colors.

The 2D VMT liquid crystalline dispersion pixels can be assembled into an array with interactive display functions. For instance, we designed and fabricated a prototypical displayable billboard for potential interactive large-screen that used in smart architectures (see Methods and Fig. S29). A hash addressing algorithm was developed to determine the specific pixels that will be turned on in the billboard by controlling corresponding relays (see Fig. S30 and Table S3). The displayable billboard can be remotely controlled by a smartphone, and showed letters ‘T’, ‘H’ and ‘U’ (see Fig. 3d and Video S2). By integrating it with a hand-tracking module, it can automatically display numbers such as ‘1’, ‘2’ and ‘3’ after capturing the gestures of the human hand (see Fig. 3e and Video S3). The array also had some other attractive performance parameters, such as high uniformity, low power consumption and negligible ultraviolet degradation. For instance, a display with 97% consistency among all pixels was found by luminance tests (Fig. S31). For an array where the backlight was supplied from the reflection of a solar beam, the power consumption was ∼123 W m^−2^ and was reduced by 35% compared with that using a light emitting diode screen as a backlight (∼189 W m^−2^) (see Text S12 and Table S4). The energy consumption of the 2D VMT liquid crystalline dispersion display (100–200 W m^−2^) was comparable to commercially available LC display techniques [44]. We performed a long-term stability test of the 2D VMT liquid crystalline dispersion, where its display gave a small decay of <1% for both outdoor (after 1000 hours under sunlight) and indoor tests (after 8000 hours) (Fig. 3f). In contrast, organic LCs are likely to undergo photo-degradation after exposure to UV irradiation for hours [45,46], making its outdoor use challenging. In addition, a transient test was performed to examine the dynamic process and the response time of the device. Fig. S32 and Text S13 collectively show that both the rising and falling edges agreed well with theoretical exponential processes, when polydispersity is taken into account. The characteristic time of rising edge and that of falling edge were determined to be 0.35 s and 2.05 s, respectively. Finally, we compared the 2D VMT liquid crystalline dispersion display with other electro-optical materials and electrochromic techniques in a radar plot (see Methods, Fig. 3g and Table S5). It can be seen that different materials had their own characteristics. For example, compared with electrochromic polymers and metal oxides, the 2D VMT liquid crystalline device had a small operational electric field and satisfactory UV stability. However, the second-level response time is relatively long compared with commercial LCs. The response time was still comparable with the products of switchable solar shading windows, privacy glazing shutters, and dynamic billboards, etc. In these cases, 2D VMT liquid crystalline dispersion may serve as a promising complement to existing materials and relevant techniques. Above all, the power-saving, ultraviolet-stable, and integration compatible 2D VMT liquid crystalline dispersion devices offer clear opportunities for outdoor LC optics in the future.

## CONCLUSION

We have prepared a 2D VMT liquid crystalline dispersion with an anomalously large electro-optical Kerr coefficient of 3.0 × 10^−4^ m V^−2^. Our work provides inspiring evidence on 2D ferroelectrics in layered natural minerals, which sheds light on the scalable production of van der Waals ferroelectric materials. These results also indicate opportunities to design advanced inorganic LCs or LC-like systems with an ultra-high electro-optical performance by preparing 2D materials from bulk ferroelectric materials.

## METHODS

The 2D VMT liquid crystalline dispersion was prepared by a cation-exchange method. The morphology of 2D VMT was examined by an atomic force microscope and a transmission electron microscope. The stability of the 2D VMT liquid crystalline dispersion was examined by a zeta potential analyzer. The rheological behavior was studied using a rheometer.

The ferroelectricity of 2D VMT was examined by a PFM measurement, which was carried out using an atomic force microscope in a Dual AC Resonance Tracking mode. The polarization versus voltage hysteresis loops were obtained using a ferroelectric tester. The first principles calculations were performed to analyze the ferroelectricity of VMT.

A quartz cuvette, with a square cross-section and 10 mm between opposite internal faces (10 mm × 10 mm × 45 mm), was filled with the 2D VMT liquid crystalline dispersion and used for electro-optical measurements. Two parallel copper plates were placed on the other internal faces of the cuvette and were used as counter electrodes to supply an electric field perpendicular to the optical path. A generator and an amplifier were combined to provide an electric field. A power meter, a polarimeter, or a digital camera were used as detectors as required. Birefringence was determined by recording the azimuth angle and ellipticity of the transmitted light.

A square quartz cuvette with a display area of 1.4 inches was fabricated to be a proof-of-concept displayable pixel. For a backlight-free pixel, an aluminum mirror was put behind it. We placed red, green or blue filters between the mirror and the pixel to generate colors from the natural light. A screen displaying standard red with RGB color code of (255, 0, 0), green (0, 255, 0), blue (0, 0, 255) and colored sunflower pictures were used as a backlight when needed. A printed circuit board was designed to assemble 15 pixels. A home-developed hash addressing algorithm was pre-written to an Arduino chip, where the Arduino chip was used to control the relays and the connected pixels. A wireless communication module and a hand-tracking module were used to realize the remote control of the pixels by smartphone or human gestures. The device performances, such as operational stability, luminance, and response time were evaluated.

All the details are given in the Supplementary information.

## Supplementary Material

nwae108_Supplemental_File

## Data Availability

The data that support the findings of this study are available within the paper and the Supplementary information. Other relevant data are available from the corresponding authors on reasonable request. Source data are provided with this paper.
