# Supplementary material for: An inorganic liquid crystalline dispersion with 2D ferroelectric moieties
Source: Natl Sci Rev. 2024 Mar 21;11(5):nwae108. doi: 10.1093/nsr/nwae108 (PMC11055536; doi:10.1093/nsr/nwae108)
Supplement: nwae108_Supplemental_File [file nwae108_supplemental_file.zip › Supplementary data.pdf]

# Supplementary Information for *National Science Review*

## An Inorganic Liquid Crystalline Dispersion with 2D Ferroelectric Moieties


Ziyang Huang[1,†], Zehao Zhang[1,†], Rongjie Zhang[1], Baofu Ding[1,2,*], Liu Yang[3], Keyou Wu[1], Youan Xu[1,4], Gaokuo Zhong[5], Chuanlai Ren[5], Jiarong Liu[1], Yugan Hao[1], Menghao Wu[3], Teng Ma[6] and Bilu Liu[1,*]

[1]Shenzhen Graphene Centre, Shenzhen Key Laboratory of Advanced Layered Materials for Value-added Applications, Tsinghua−Berkeley Shenzhen Institute and Institute of Materials Research, Tsinghua Shenzhen International Graduate School, Tsinghua University, Shenzhen 518055, China;

[2]Institute of Technology for Carbon Neutrality, Faculty of Materials Science and Engineering, Shenzhen Institute of Advanced Technology, Chinese Academy of Sciences, Shenzhen 518055, China;

[3]School of Physics and Institute for Quantum Science and Engineering, School of Chemistry and Institute of Theoretical Chemistry, Huazhong University of Science and Technology, Wuhan 430074, China;

[4]Xi'an Research Institute of High Technology, Xi’an 710025, China;

[5]Shenzhen Institute of Advanced Technology, Chinese Academy of Sciences, Shenzhen 518055, China;

[6]Department of Applied Physics, Hong Kong Polytechnic University, Hong Kong, China

[*]**Corresponding authors**. E-mails: bf.ding@siat.ac.cn; bilu.liu@sz.tsinghua.edu.cn

[†]Equally contributed to this work.

**This Supplementary Information includes:**

Methods

Texts S1-13

Figures S1-32

Tables S1-5

Captions for Videos S1-3

Supplementary References 1-59

**Contents of Supplementary Texts, Figures, and Tables:**

Text S1 | Calculations of the birefringence based on the azimuth angle and ellipticity of polarization. ........................................................................................................10

Text S2 | Intrinsic parameters that determine the Kerr coefficient $\boldsymbol{K}$.........................11

Text S3 | Experimental determination of the optical anisotropy factor $\Delta\boldsymbol{g}$ of 2D VMT liquid crystalline dispersion from electro-birefringence tests....................................12

Text S4 | Frequency dependence of the Kerr coefficient $\boldsymbol{K}$ and the determination of excess electrical polarizability anisotropy $\Delta\boldsymbol{\alpha}$ and the inherent dipole $\boldsymbol{\mu}$ of 2D VMT liquid crystalline dispersion.........................................................................12

Text S5 | Theoretical phase transition of 2D VMT liquid crystalline dispersion.........13

Text S6 | Theoretical calculations of the origin of the inherent electric dipole and the ferroelectricity.........................................................................................14

Text S7 | Estimation of the balance distance of 2D VMT platelets based on the Derjaguin–Landau–Verwey–Overbeek theory...........................................................15

Text S8 | Theoretical calculation of the optical anisotropy factor $\Delta\boldsymbol{g}$ of 2D VMT liquid crystalline dispersion. ........................................................................................15

Text S9 | Theoretical calculation of the excess electrical polarizability anisotropy $\Delta\boldsymbol{\alpha}$ of 2D VMT liquid crystalline dispersion....................................................................16

Text S10 | Experimental determination of the inherent dipole $\boldsymbol{\mu}$ of 2D VMT liquid crystalline dispersion from polarization tests. ...........................................................18

Text S11 | Giant Kerr effect enabled by the geometrical anisotropy factor $\boldsymbol{\gamma}$ and intrinsic polarization $\boldsymbol{P}$. ........................................................................................ 19
Text S12 | Estimation of the energy consumption of 2D VMT liquid crystalline dispersion devices. ........................................................................................ 20
Text S13 | Transient test and response time of 2D VMT liquid crystalline dispersion devices ........................................................................................ 20

Fig. S1 | Bulk VMT and its atomic structure. ............................................................ 23
Fig. S2 | Preparation of 2D VMT dispersion. ............................................................ 24
Fig. S3 | Electrostatic shielding effect of ions. ............................................................ 25
Fig. S4 | Characterization of 2D VMT liquid crystalline dispersion. .......................... 26
Fig. S5 | Phase transition behavior of 2D VMT liquid crystalline dispersion. ............ 27
Fig. S6 | Global observation of flow-induced birefringence of 2D VMT liquid crystalline dispersion with a volume fraction of 0.08 vol%. ...................................... 28
Fig. S7 | Local observation of flow-induced birefringence of 2D VMT liquid crystalline dispersion with a volume fraction of 0.08 vol%. ....................................................... 29
Fig. S8 | SAXS of 2D VMT liquid crystalline dispersion with volume fractions of $8.0\times10^{-4}$ and 1.2 vol%. ........................................................................................ 30
Fig. S9 | Rheological tests of 2D VMT liquid crystalline dispersion. ......................... 31
Fig. S10 | Schematics of the polarization-dependent transmittance. ........................... 32
Fig. S11 | A POM image showing parallel textures. .................................................... 33
Fig. S12 | Schematics of the Kerr experiments. ........................................................... 34
Fig. S13 | Polarization ellipse of 2D VMT liquid crystalline dispersion with a volume fraction of 0.08 vol%. ........................................................................................ 35
Fig. S14 | Saturated birefringence of 2D VMT liquid crystalline dispersion. ............. 36
Fig. S15 | Morphology characterization on 2D VMT liquid crystalline dispersion..... 37
Fig. S16 | 2D VMT coated on a conducting Au substrate for PFM measurement....... 38
Fig. S17 | Polarization switching of 2D VMT by PFM. .............................................. 39
Fig. S18 | Surface potential of 2D VMT by KPFM. ................................................... 40
Fig. S19 | 2D VMT laminate film used for the polarization test................................. 41

Fig. S20 | Polarization *versus* voltage hysteresis loops of an assembled 2D VMT laminate film at different frequencies. ........................................................................42
Fig. S21 | Crystalline structure of 2D VMT and the origin of ferroelectricity............43
Fig. S22 | PFM phase maps of 2D VMT. ..................................................................44
Fig. S23 | POM images of 2D VMT dispersion with a volume fraction of 2.0 vol%..45
Fig. S24 | Theoretical interaction potential between 2D VMT platelets......................46
Fig. S25 | AFM images and statistics of 2D VMT liquid crystalline dispersion in which 2D VMT has different lengths. ........................................................................47
Fig. S26 | Kerr coefficients of 2D VMT liquid crystalline dispersion in which 2D VMT has different geometrical anisotropy factor.................................................................48
Fig. S27 | Dependence of the Kerr coefficients with temperature. ..............................49
Fig. S28 | Optical band gap of 2D VMT liquid crystalline dispersion and its transmittance. ..........................................................................................................50
Fig. S29 | Custom-made device array integrating 15 displayable macroscopic pixels. ...............................................................................................................................51
Fig. S30 | A mechanism schematic of the hash addressing algorithm. ........................52
Fig. S31 | Luminance test on the pixels of the gesture capture array...........................53
Fig. S32 | Transient electric birefringence test of 2D VMT liquid crystalline dispersion device. ..................................................................................................................54

Table S1 | Azimuth angle, ellipticity, and birefringence of 2D VMT liquid crystalline dispersion with a volume fraction of 0.08 vol%. ........................................................55
Table S2 | Comparison of the Kerr coefficient $\boldsymbol{K}$ of 2D VMT liquid crystalline dispersion with other electro-optical Kerr media........................................................56
Table S3 | Pseudo code used for the displayable array in the Figure 3 of the main text. ...............................................................................................................................59
Table S4 | Estimation of energy consumption of the displayable array based on 2D VMT liquid crystalline dispersion. ...............................................................................60
Table S5 | Comparison of 2D VMT liquid crystalline dispersion devices with other electrochromic techniques. ..........................................................................................61

**Other Supplementary Information for this manuscript includes the following:**

Videos S1-3

# Methods

## Preparation of 2D VMT liquid crystalline dispersion and characterization

Vermiculite (VMT, sizes: 2-3 mm, Sigma-Aldrich, USA), sodium chloride (NaCl, purity >99.5%, Shanghai Macklin Biochemical Co., Ltd., China) and lithium chloride (LiCl, purity >99.0%, Shanghai Macklin Biochemical Co., Ltd., China) were used without any treatment. The 2D VMT liquid crystalline dispersion was obtained following a two-step ion-exchange method. The VMT was immersed in 100 mL of saturated NaCl solution (26.5 wt%) and stirred on a hot stage at 80 ℃ for 24 h. The solution was collected and washed with deionized water repeatedly to remove residual salts. The $Na^+$ exchanged VMT precursor was immersed in 100 mL 2 M LiCl solution and stirred on a hot stage at 80 ℃ for another 24 h. The products were repeatedly washed with deionized water until the ionic strength went below $10^{-4}$. The unexfoliated VMT was removed by ultracentrifugation. The morphology of the 2D VMT was investigated by an atomic force microscope (AFM, tapping mode, Cyper ES, Oxford Instruments, USA) and a transmission electron microscope (TEM, 120 kV, Spirit T12, FEI, USA). The stability of the 2D VMT liquid crystalline dispersion was examined by a zeta potential analyzer (Zetasizer Nano-ZS90, Malvern, UK). Small angle X-ray scattering data was obtained using a Nanostar SAXS system, Bruker, Germany. The rheological behavior was studied using a rheometer (MCR-302, Anton Paar, Austria).

## PFM and polarization measurements

2D VMT was coated on a conducting Au substrate for PFM measurement using a Langmuir−Blodgett method. The 2D VMT dispersion was vacuum-filtrated to remove the deionized water, and 2D VMT remaining on the filtration substrate was re-dispersed in a mixed solvent of methanol and chloroform with a ratio of 1:1. Then, it was carefully dropped onto the surface of a water bath, in which an Au substrate had been submerged. Two baffles on opposite sides of the bath were steadily moved towards each other to achieve a tight arrangement of 2D VMT on the water surface until the

surface pressure reached 10 mN $m^{-1}$. The Au substrate was pulled up at a rate of 1 mm $min^{-1}$, where 2D VMT adhered to its surface. PFM measurements were carried out by atomic force microscopy (Cyper ES, Oxford Instruments, USA) in a Dual AC Resonance Tracking mode. PFM amplitude and phase loops were recorded using the spectroscopy channel. The heart and box-in-box patterns were written by applying DC poling voltages of -10 V and 10 V at different positions with a sweep rate of 1.92 Hz. A conducting tip with a resonance frequency of 350-400 kHz was driven by a DC voltage in the range of 0.8-1.5 V to scan the sample after the writing process. An additional voltage of 2.2-2.8 V was applied to the tip to eliminate the surface electrostatic effect of 2D VMT. 50 mL of a 2D VMT Kerr dispersion with a volume fraction of 0.04 vol% was used to prepare an assembled 2D VMT laminate film by vacuum filtration to characterize the macroscopic polarization. Polarization *versus* voltage hysteresis loops were obtained using a ferroelectric tester (Precision Premier II, Radiant Technologies, USA). The testing frequencies were 0.1 kHz and 100 kHz.

**Ferroelectricity calculations**

The density-function-theory calculations were performed with the use of projected augmented wave potential implemented Vienna Ab initio Simulation Package (VASP 5.4.4) code [1-3]. The exchange-correlation interactions were described within the generalized gradient approximation using the Perdew-Burke-Ernzerhof form [4]. The DFT-D3 method with Becke-Johnson damping function was used to describe the van der Waals interactions [5, 6]. Cut-off energy of 520 eV was set for the plane-wave basis, and the Brillouin zone was sampled with $6\times4\times1$ k-points using the Monkhorst-Pack scheme [7]. The shape and volume of unit cell were fully optimized with the convergence criteria being $1\times10^{-6}$ eV for the energy difference in the electronic self-consistent iteration and $1\times10^{-3}$ eV $Å^{-1}$ for the residual force on all atoms. The vacuum layers were set as 23 Å so that the interaction between two neighboring bilayer can be neglected. The Berry phase method was used to compute electrical polarizations [8], and the climbing image nudged elastic band method was adopted to compute the ferroelectric switching pathway [9].

**Electro-optical measurements**

A quartz cuvette, with a square cross-section and 10 mm between opposite internal faces (10 mm × 10 mm × 45 mm), was filled with the 2D VMT liquid crystalline dispersion and used for electro-optical measurements. Two parallel copper plates were placed on the other internal faces of the cuvette and were used as counter electrodes to supply the electric field perpendicular to the optical path. A generator (AFG 3102C, Tektronix Inc., USA) and an amplifier (ATA-2082, Aigtek Co. Ltd., China) were combined to provide an electric field with frequencies of 10-1000 kHz. A polarizer (GL10-A, Thorlabs Inc., USA) was placed in the incident light path. Optical images were taken by a polarized optical microscope (Imager A2m, Carl Zeiss, Germany) or a Nikon D7000 digital camera with a lens of AF-S DX Nikkor 18-140 mm f/3.5–5.6G ED VR. A 450 nm laser was used as the incident light. A power meter (PM 200, Thorlabs Inc., USA) or a polarimeter (PAX1000, Thorlabs Inc., USA) was put in the output optical path as a detector. For polarization-dependent transmittance, the polarizer was rotated for 360 degrees, and the intensity of transmitted light was recorded by a power meter. For quantitative measurement of the electro-birefringence $\Delta n$, the polarization direction was set at 45 degrees to the direction of the electric field. The polarization signal recorded by the polarimeter can be converted to $\Delta n$ using $\Delta n = \frac{\lambda}{2\pi L}\left\{arctan[tan(\theta) \times tan(\eta)] + arctan\left[\frac{tan(\eta)}{tan(\theta)}\right]\right\}$, where $\lambda$ is the wavelength, $L$ the optical path, $\theta$ the azimuth angle, and $\eta$ the ellipticity. A detailed derivation was given in Text S1.

**Fabrication of prototypical devices**

A square quartz cuvette with a display area of 1.4 inches was fabricated to be a proof-of-concept displayable pixel. The polarizers and electrodes were set as mentioned earlier in 'Electro-optical measurement' section. For a backlight-free pixel, an aluminum mirror was put behind it. We placed red, green or blue filters between the mirror and the pixel to generate colors from the natural light. A screen displaying

standard red with a RGB color code of (255, 0, 0), green (0, 255, 0), blue (0, 0, 255) and colored sunflower pictures were used as a backlight when needed. An electric field of $4 \times 10^3$ V $m^{-1}$ was applied to turn on the pixel, which is black without an external electric field. To integrate the pixels, we designed a printed circuit board, where 15 pixels were assembled on it, as shown in Fig. S29. An open-source Arduino chip (Arduino Mega, Creative Commons) was used to dynamically control the relay array, to which all the pixels were connected. A home-developed hash addressing algorithm was pre-written on the Arduino chip, which was responsible for finding the relays to be turned on when receiving a signal. A wireless communication module remotely controlled by software was connected to the billboard if needed. A hand-tracking module (Leap Motion Controller, Ultraleap Limited, USA) was used to capture the gestures of a human hand. For both cases, the electric field was pre-set as 0, $4 \times 10^3$ or $8 \times 10^3$ V $m^{-1}$. The electric field was applied when the controller clicked the pixel icons shown in the smartphone software (Blinker, Diandeng technology, China) or the hand-tracking module capture the gesture of the controller. All images were taken by a Nikon D7000 digital camera with a lens of AF-S DX Nikkor 18–140 mm f/3.5–5.6G ED VR.

**Evaluation of device performance**

Operational stability was monitored by recording the luminance of the pixel for long-term operation. The luminance was measured by a spectroradiometer (SpectraDuo PR-680, Photo Research Inc., USA). For an indoor test, we kept the 2D VMT liquid crystalline dispersion in a dark place. For an outdoor test, we exposed it to sunlight, where we estimated an exposure time of 8 hours per day. The response time ($t_R$) was examined by a transient test and the rising edge and falling edge both follow an exponential process. Here, we used the characteristic time of rising edge to represent the response time $t_{R.}$ The optical density ($\Delta OD$) was calculated according to $\Delta OD = log\frac{T_{on}(\lambda_{max})}{T_{off}(\lambda_{max})}$ , where $T_{on}(\lambda_{max})$ and $T_{off}(\lambda_{max})$ are respectively transmittance of the pixel at on and off states at the peak wavelength. The Michelson contrast was used following $CR = \frac{T_{on}^{int} - T_{off}^{int}}{T_{on}^{int} + T_{off}^{int}}$. Here, $T_{on}^{int}$ and $T_{off}^{int}$ are integrated

transmittance in visible spectral region at off and on states.

## Supplementary Text

### Text S1 | Calculations of the birefringence based on the azimuth angle and ellipticity of polarization.

A birefringent media can change the linearly polarized light to an elliptically polarized light, where the polarization state is represented by an ellipse. As for an electromagnetic wave, the electric field of a polarized light $E^{light}$ can be written as

$$E^{light}(t) = E_a^{light}\hat{a}\cos\omega t - E_b^{light}\hat{b}\sin\omega t.$$

The $\hat{a}$ and $\hat{b}$ are two basis vectors along the major and minor axis of the ellipse. The $E_a^{light}$ and $E_b^{light}$ are amplitudes of the electric field intensity in $\hat{a}$ or $\hat{b}$ direction. Changing the basis vectors to $\hat{x}$ and $\hat{y}$, we get

$$\begin{bmatrix}\hat{x}\\ \hat{y}\end{bmatrix} = \begin{bmatrix}\cos\theta & -\sin\theta\\ \sin\theta & \cos\theta\end{bmatrix}\begin{bmatrix}\hat{a}\\ \hat{b}\end{bmatrix}.$$

Therefore,

$$E^{light}(t) = \left(E_a^{light}\cos\theta\cos\omega t + E_b^{light}\sin\theta\sin\omega t\right)\hat{x}$$
$$+ \left(E_a^{light}\sin\theta\cos\omega t - E_b^{light}\cos\theta\sin\omega t\right)\hat{y}$$

$$E^{light}(t) = E_x^{light}\cos(\omega t + \delta_x)\,\hat{x} + E_y^{light}\cos\left(\omega t + \delta_y\right)\hat{y}$$

$$E(t) =$$
$$E_x^{light}(\cos\delta_x\cos\omega t - \sin\delta_x\sin\omega t)\,\hat{x} + E_y^{light}\left(\cos\delta_y\cos\omega t - \sin\delta_y\sin\omega t\right)\hat{y}.$$

Since $E_x^{light}\cos\delta_x = E_a^{light}\cos\theta$ and $-E_x^{light}\sin\delta_x = E_b^{light}\sin\theta$, then we get

$$-\frac{\sin\delta_x}{\cos\delta_x} = \frac{E_b^{light}}{E_a^{light}}\cdot\frac{\sin\theta}{\cos\theta}, \text{ and}$$

$$\tan\delta_x = -\tan\eta\cdot\tan\theta,$$

where $\theta$ is the azimuth angle and $\eta$ is the ellipticity. Similarly, it comes

$$\tan\delta_y = \frac{\tan\eta}{\tan\theta}.$$

Therefore, the phase retardation $\delta$ can be calculated by following formula,

$$\delta = \delta_y - \delta_x = \arctan[\tan(\theta)\times\tan(\eta)] + \arctan\left[\frac{\tan(\eta)}{\tan(\theta)}\right].$$

In addition, $\Delta n = \frac{\lambda}{2\pi L}\delta$ ($\lambda$ is the wavelength and $L$ is the optical path), so that

$$\Delta n = \frac{\lambda}{2\pi L}\left\{\arctan[\tan(\theta) \times \tan(\eta)] + \arctan\left[\frac{\tan(\eta)}{\tan(\theta)}\right]\right\}.$$

**Text S2 | Intrinsic parameters that determine the Kerr coefficient $K$.**

As a quadratic electro-optical effect, the Kerr effect indicates that birefringence $\Delta n$ is proportional to the square of electric field strength $E^2$ with a Kerr coefficient $K$, shown in Eq. S1.

$$\Delta n = K\lambda E^2 \text{ (Eq. S1)}$$

When a Kerr medium is dispersed in a solvent, three parameters contribute to $\Delta n$, namely specific birefringence $\Delta n^p$, concentration (volume fraction) of the medium $\varphi$ and orientational order parameter $O(E)$ [10, 11]. This gives

$$\Delta n = \Delta n^p \varphi O(E) \text{ (Eq. S2).}$$

$\Delta n^p$ originates from the differences in dielectric properties between the dispersed Kerr medium and the solvent, as well as the resulting heterogeneity of refractive indices, which is independent of the ordered state induced by an electric field. The optical anisotropy factor $\Delta g$, namely, the principle excess optical polarizabilities per unit volume, is commonly used to describe $\Delta n^p$ using Eq. S3, where $n$ is the refractive index, the subscript $s$ represents the solvent, and $\varepsilon_0$ the vacuum permittivity [12-14]. A detailed discussion of $\Delta g$ is provided in Text S3 and Text S8.

$$\Delta n^p = \frac{\Delta g}{2n_s\varepsilon_0} \text{ (Eq. S3)}$$

Orientational order parameter $O(E)$ provides all the information about the orientation of dispersed particles driven by potential energy $U$ in an electric field. O'Konski et al. have shown that $U$ is determined by both the inherent dipole moment $\mu$ of dispersed particles and the anisotropy of excess electrical polarizabilities of particles beyond the isotropic electrical polarizability of dispersant itself $\Delta\alpha$ [10, 11], where

$$U = \mu E cos\theta_{LC-E} + \frac{1}{2}\Delta\alpha E^2 cos^2\theta_{LC-E} \text{ (Eq. S4),}$$

and $\theta_{LC-E}$ the angle between the direction of $E$ and the preferred axis of

polarizability of the particles. A detailed discussion on excess electrical polarizability anisotropy $\Delta\alpha$ is provided in Text S9. The angular distribution function $A(\theta_{LC-E})$ then gives,

$$A(\theta_{LC-E}) = \frac{exp\left(-\frac{U}{k_BT}\right)}{\int_0^{\pi} \exp(-\frac{U}{k_BT})\cdot 2\pi sin\theta_{LC-E} d\theta_{LC-E}}$$

$$= \frac{exp\left(-\frac{\mu E}{k_BT}cos\theta_{LC-E} - \frac{\Delta\alpha}{2k_BT}E^2cos^2\theta_{LC-E}\right)}{2\pi\int_{-1}^{1} exp\left(-\frac{\mu E}{k_BT}cos\theta_{LC-E} - \frac{\Delta\alpha}{2k_BT}E^2cos^2\theta_{LC-E}\right)dcos\theta_{LC-E}} \text{ (Eq. S5),}$$

where $k_B$ is the Boltzmann constant and $T$ the Kelvin temperature. Following the basic equation of orientational order parameter $O(E)$ in Eq. S6, we find $O(E)$ can be expressed as Eq. S7, under the condition of $E \rightarrow 0$.

$$O(E) = \pi\int_0^{\pi}(3cos^2\theta_{LC-E} - 1)A(\theta_{LC-E})sin\theta_{LC-E}\, d\theta_{LC-E} \text{ (Eq. S6)}$$

$$O(E \rightarrow 0) = \frac{1}{15}\left(\frac{\Delta\alpha}{k_BT}E^2\right) + \frac{1}{15}\left(\frac{\mu}{k_BT}E\right)^2 \text{ (Eq. S7)}$$

The conclusion is that $K$ is jointly determined by $\Delta g$, $\Delta\alpha$ and $\mu$ according to Eq. S8. This is further simplified to Eq. S9 by designating $Z_1 = \frac{\varphi}{30n_s\varepsilon_0\lambda}$ and $Z_2 = \frac{1}{k_BT}$.

$$K = \frac{\varphi\Delta g}{30n_s\varepsilon_0\lambda}\left[\frac{\Delta\alpha}{k_BT} + \left(\frac{\mu}{k_BT}\right)^2\right] \text{ (Eq. S8)}$$

$$K = Z_1Z_2\Delta g(\Delta\alpha + Z_2\mu^2) \text{ (Eq. S9)}$$

**Text S3 | Experimental determination of the optical anisotropy factor $\Delta g$ of 2D VMT liquid crystalline dispersion from electro-birefringence tests.**

$\Delta g$ is experimentally determined by the measurement of saturated birefringence $\Delta n_{sat}$, where the saturated orientational order parameter $O_{sat} \rightarrow -0.5$ [15, 16]. In that case, Eq. S2 is reorganized to Eq. S10.

$$2\Delta n_{sat} = -\Delta n^p\varphi \text{ (Eq. S10)}$$

From the linear fit in Fig. S14b, $\Delta n^p$ is determined to be $-1.0 \times 10^{-1}$ and $\Delta g = -2.3 \times 10^{-12}\ C^2J^{-1}m^{-1}$.

**Text S4 | Frequency dependence of the Kerr coefficient $K$ and the determination of excess electrical polarizability anisotropy $\Delta\alpha$ and the inherent dipole $\mu$ of**

**2D VMT liquid crystalline dispersion.**

Figure 1h shows a dependence of $K$ with the frequency $f$ of the applied electric field. The decay of $K$ may be due to both $\Delta\alpha$ and $\mu$ [17, 18], according to

$$K = \frac{\Delta g\varphi}{30 n_s \varepsilon_0 \lambda}\left[\frac{\Delta\alpha}{k_B T}\frac{1}{1+\left(f/f_c^{EDL}\right)^2} + \left(\frac{\mu}{k_B T}\right)^2 \frac{1}{1+\left(f/f_c^{dipole}\right)^2}\right] \text{ (Eq. S11).}$$

The critical frequency $f_c^{EDL}$, representing the contribution of $\Delta\alpha$, is predicted to be 5.0 MHz based on Maxwell-Wagner-O'Konski theory following Eq. S12. [11, 15, 19] A detailed discussion of Maxwell-Wagner-O'Konski theory is in Text S9.

$$f_c^{EDL} \approx \frac{1}{8}\frac{\langle H\rangle}{\langle D\rangle}\frac{C_{p,2}}{\varepsilon_0\varepsilon_e} \text{ (Eq. S12)}$$

Hence, the frequency dispersion in the range of 10 to 1000 kHz is ascribed to $\mu$, where $f_c^{dipole}$ is determined to be 140 kHz. Eq. S11 then indicates that, $\frac{1}{1+\left(f/f_c^{EDL}\right)^2}$ is approximately 1 in the experimental range of 10 to 1000 kHz, while $K$ depends on the item of the inherent dipole. Using Eq. S11, we get $\Delta\alpha = -9.3 \times 10^{-27}\, F\, m^2$ and $|\mu| = 1.7 \times 10^{-23}\, C\, m.$

**Text S5 | Theoretical phase transition of 2D VMT liquid crystalline dispersion.**

We calculate the critical volume fraction for the nematic phase transition using the Onsager excluded volume theory [11, 20, 21]. Given that the overall length distribution of dispersed 2D VMT follows a Gaussian distribution, the dimensionless density $\rho_N$ at the biphasic-nematic phase transistion $\varphi_{B-N}$ is estimated to be $\rho_N\langle D\rangle^3 = 3.98 + 15.82\sigma^2$, where $\sigma$ is the standard deviation. By taking the experimental data of average length $\langle D\rangle = 2.6\,\mu m$, weighted average height $\langle H\rangle = 1.7\,nm$ and $\sigma = 0.78$ from Fig. 2a-b and Fig. S15b, we convert the dimensionless density $\rho_N$ to the critical volume fraction according to Eq. S13.

$$\varphi_{B-N} = \frac{\pi\rho_N\langle D\rangle^2\langle H\rangle}{4} = \frac{\pi\langle H\rangle}{4}\cdot\frac{\rho_N\langle D\rangle^3}{\langle D\rangle}\cdot\frac{1+\sigma^2}{1+3\sigma^2} = \frac{\pi(1+\sigma^2)(3.98+15.82\sigma^2)}{4(1+3\sigma^2)}\frac{\langle H\rangle}{\langle D\rangle} \text{ (Eq. S13)}$$

The result gives that the critical volume fraction for the nematic phase transition is approximately 0.40 vol%.

**Text S6 | Theoretical calculations of the origin of the inherent electric dipole and the ferroelectricity.**

We perform a simulation on a monolayer VMT by first principles calculations, which basically possess a formula of $(Mg_6Si_8O_{20})(OH)_4$ as shown in Fig. S21a. As elemental substitution commonly takes place in VMT, we consider the case that Mg ion is substituted by Al or Fe ion in the middle $MgO_2$ sheet. These substitution does not deviate from the center of octahedron after structural optimization. Meanwhile, every such substitution will give rise to a proton vacancy in the middle layer to maintain the closed-shell electron number for chemical stability of the system. For a monolayer VMT, approximately 1/3 of Mg ions are substituted in the middle $MgO_2$ sheet (following the electron probe microanalysis results [16]). The vertical polarization is estimated to be respectively 0.17 or 0.12 e* Å by Al or Fe substitution, and the horizontal polarization is estimated to be respectively 1.30 or 0.82 e* Å by Al or Fe substitution. Therefore, there exist both in plane and out-of-plane polarization, and the in-plane one is about 7 times larger than the out-of-plane one. According to our calculations in Fig. S21b and Fig. S21c, the switching barrier for each proton is estimated to be around 2.3 eV (for both Al and Fe substitution, within a unit cell containing 41 atoms), in which the ion displacements both in-plane and out-of-plane mainly involve one proton, one Al/Fe ion and three O atoms during switching, implying a much higher barrier compared with previously reported proton-transfer ferroelectrics (~50 meV) [22], as well as most perovskite ferroelectrics (~100 meV) and sliding ferroelectrics (~meV) [23]. The calculation that indicates 2D VMT possesses both an in-plane and out-of-plane polarization agrees well with the experimental observation (Fig. S22). Distinct from sliding ferroelectricity with robustness ensured by the in-plane rigidity of 2D materials enforcing all dipoles aligned to the same direction [24], herein the dipoles induced by proton vacancies are mostly independent and the robustness of ferroelectricity can be ensured simply by the high switching barrier. Due to the high barrier and the nuclear quantum effect of protons [25, 26], only a small portion of protons can tunnel across the barrier, which may explain why the measured

polarization is much smaller than the predicted maximum value, but increases rapidly as larger electric field is applied and the probability for protons tunneling the barrier is greatly enhanced (Fig. 2g). These results qualitatively agree with the experimental results.

**Text S7 | Estimation of the balance distance of 2D VMT platelets based on the Derjaguin–Landau–Verwey–Overbeek theory.**

Derjaguin–Landau–Verwey–Overbeek theory is usually used to explain the balance between interparticle van der Waals attraction force and the electrostatic repulsive in a colloidal system. The potential energy gives that $P = P_A + P_R = -\frac{H_A}{12}\left[\frac{1}{d^2} + \frac{1}{(d+2\langle H\rangle)^2} - \frac{2}{(d+\langle H\rangle)^2}\right] + \frac{64N_A I k_B T}{\kappa}\left[tanh\left(\frac{e\zeta}{4k_B T}\right)\right]^2 e^{-\kappa d}$ , where $H_A$ is the Hamaker constant (we estimate a Hamaker constant ~ $2\times10^{-20}$ J following reports for other clay minerals [27]), $\langle H\rangle$ is the average height of 2D VMT, $N_A$ is the Avogadro constant, $I$ is the ionic strength, $\kappa$ is the reciprocal of the Debye length $\kappa^{-1}$, $e$ is the charge of an electron, and $\zeta$ is the Zeta potential. For 2D VMT liquid crystalline dispersion, the ionic strength is approximately $1\times10^{-4}$ and the Debye length is determined to be 31 nm following $\kappa^{-1} = \sqrt{\frac{\varepsilon_0\varepsilon_S k_B T}{2N_A I e^2}}$. A secondary minimum of the potential curve emerges at a distance of 726 nm for a 2D VMT liquid crystalline dispersion

**Text S8 | Theoretical calculation of the optical anisotropy factor $\Delta g$ of 2D VMT liquid crystalline dispersion.**

$\Delta g$ arises from both the intrinsic anisotropy of the refractive index of the particles and another geometrical-optical item relating to the excess refractive indices between the particle and the solvent [12-14]. Given that the in-plane refractive indices are isotropic and the anisotropy is between the out-of-plane and in-plane properties, $g$ factor and $\Delta g$ are respectively written as Eq. S14 and Eq. S15, where the subscript $p$ represents the particle, $s$ the solvent, $1$ the direction parallel to the normal director of 2D VMT (out-of-plane axis), $2$ the direction perpendicular to the normal director of 2D VMT (in-plane axis), $\varepsilon$ the relative permittivity with an approximation of

$\varepsilon = n^2$, and $F$ the depolarization factor.

$$g_k = 4\pi\varepsilon_0 \frac{n_{p,k}^2 - n_s^2}{4\pi + \frac{n_{p,k}^2 - n_s^2}{n_s^2} \cdot F_k}, k = 1, 2 \quad \text{(Eq. S14)}$$

$$\Delta g = g_1 - g_2 = \varepsilon_0 \varepsilon_s \frac{\varepsilon_s(\varepsilon_{p,1} - \varepsilon_{p,2}) + (\varepsilon_{p,1} - \varepsilon_s)(\varepsilon_{p,2} - \varepsilon_s)\frac{(F_{p,2} - F_{p,1})}{4\pi}}{\left(\varepsilon_s + (\varepsilon_{p,1} - \varepsilon_s)\frac{F_{p,1}}{4\pi}\right)\left(\varepsilon_s + (\varepsilon_{p,2} - \varepsilon_s)\frac{F_{p,2}}{4\pi}\right)} \quad \text{(Eq. S15)}$$

For 2D VMT, we use an oblate spheroid model to give the depolarization factor $F$ [28], where $F_{p,1} \approx 4\pi\left(1 - \frac{\pi\langle H\rangle}{2\langle D\rangle}\right) = 4\pi\left(1 - \frac{\pi}{2}\gamma^{-1}\right)$, $F_{p,2} \approx 4\pi\left(\frac{\pi\langle H\rangle}{4\langle D\rangle}\right) = 4\pi\left(\frac{\pi}{4}\gamma^{-1}\right)$, and $\gamma \gg 1$ is needed.

Specifically, we take the following data to calculate $\Delta g$ with $n_{p,1} = 1.56$, $n_{p,2} = 1.58$, $n_s = 1.33$, $\langle D\rangle = 2.6\,\mu m$, and $\langle H\rangle = 1.7\,nm$. Finally, the result shows that $\Delta g = -2.2 \times 10^{-12}\, C^2 J^{-1} m^{-1}$, where the negative value indicates an in-plane easy axis following the aforementioned derivation.

To elucidate the dependence of $\Delta g$ on the geometrical anisotropy factor $\gamma$, we further simplify the Eq. S15 assuming $\varepsilon_{p,1} \approx \varepsilon_{p,2} = \varepsilon_p = 2.46$, which becomes

$$\Delta g \approx \varepsilon_0 \frac{\varepsilon_s(\varepsilon_p - \varepsilon_s)^2(2.4\gamma^{-1} - 1)}{[\varepsilon_s + (\varepsilon_p - \varepsilon_s)(1 - 1.6\gamma^{-1})][\varepsilon_s + 0.8(\varepsilon_p - \varepsilon_s)\gamma^{-1}]} \quad \text{(Eq. S16)}$$

Given that $\gamma^{-1} \ll 1$, the denominator tends to be the product of $\varepsilon_p$ and $\varepsilon_s$, and Eq. S17 shows

$$\Delta g \approx \varepsilon_0 \frac{(\varepsilon_p - \varepsilon_s)^2}{\varepsilon_p}(2.4\gamma^{-1} - 1) \quad \text{(Eq. S17)}$$

It is worth noting that Eq. S17 is only accurate when $\gamma^{-1} \ll 1$, so that $2.4\gamma^{-1} - 1 < 0$ and $\Delta g < 0$. If we designate $Z_3 = \varepsilon_0 \frac{(\varepsilon_p - \varepsilon_s)^2}{\varepsilon_p}$, we summarize that $|\Delta g|$ is negatively correlated to the reciprocal of $\gamma$, as shown in Eq. S18. A large $\gamma$ is expected for 2D LCs to approach the upper limit $Z_3$ of the optical anisotropy factor.

$$|\Delta g| \approx -2.4 Z_3 \gamma^{-1} + Z_3 \quad \text{(Eq. S18)}$$

**Text S9 | Theoretical calculation of the excess electrical polarizability anisotropy $\Delta\alpha$ of 2D VMT liquid crystalline dispersion.**

$\Delta\alpha$ is formulated based on the Maxwell-Wagner-O'Konski theory, where the induced dipole arises from the ionic clouds surrounding a charged particle, namely electrical

double layers (EDLs) [11, 29]. Eqs. S19 to S24 show the basic calculating principles of $\Delta\alpha$, which is split into two independent items, the anisotropy of permittivity $\alpha_{p,k}^{\infty}$ and the anisotropy of the effective conductivity $C$ induced by EDLs $\alpha_{p,k}^{0}$.

$$\Delta\alpha = \alpha_{p,1} - \alpha_{p,2} \quad \text{(Eq. S19)}$$

$$\alpha_{p,k} = \alpha_{p,k}^{\infty} + \frac{\alpha_{p,k}^{0} - \alpha_{p,k}^{\infty}}{1+\omega^2\tau_{p,k}^2} \quad \text{(Eq. S20)}$$

$$\alpha_{p,k}^{\infty} = \frac{\pi\langle H\rangle\langle D\rangle^2}{6}\varepsilon_0\varepsilon_s \frac{\varepsilon_{p,k}-\varepsilon_s}{\varepsilon_s+(\varepsilon_{p,k}-\varepsilon_s)\frac{F_{p,k}}{4\pi}} = V_{2D}\Delta g \quad \text{(Eq. S21)}$$

$$\alpha_{p,k}^{0} = \frac{\pi\langle H\rangle\langle D\rangle^2}{6}\varepsilon_0\varepsilon_s \frac{C_{p,k}-C_s}{C_s+(C_{p,k}-C_s)\frac{F_{p,k}}{4\pi}} \quad \text{(Eq. S22)}$$

$$\omega^2 = (2\pi f)^2 \quad \text{(Eq. S23)}$$

$$\tau_{p,k}^2 = \left[\varepsilon_0 \frac{\left(1-\frac{F_{p,k}}{4\pi}\right)\varepsilon_s+\frac{F_{p,k}}{4\pi}\varepsilon_{p,k}}{\left(1-\frac{F_{p,k}}{4\pi}\right)C_s+\frac{F_{p,k}}{4\pi}C_{p,k}}\right]^2 \quad \text{(Eq. S24)}$$

Here, $\omega$ is the circular frequency and $\tau$ the relaxation coefficient. The Maxwell-Wagner-O'Konski model assumes that the effective conductivities $C$ of a dielectric material are determined by the charge density of the EDLs $\sigma_{EDL}$, the mobility of selected ions in the EDLs $\mu_{EDL}$, and the geometrical parameters of the anisotropic particles such as $\langle D\rangle$ and $\langle H\rangle$, as shown in Eqs. S25 and S26 [15, 19].

$$C_{p,1} \approx \frac{4C_{p,1}^{EDL}}{\langle D\rangle} = \frac{4\mu_{EDL}\sigma_{EDL}}{\langle D\rangle} \quad \text{(Eq. S25)}$$

$$C_{p,2} \approx \frac{2C_{p,2}^{EDL}}{\langle H\rangle} = \frac{2\mu_{EDL}\sigma_{EDL}}{\langle H\rangle} \quad \text{(Eq. S26)}$$

Taking the typical data of $\mu_{EDL} = 3.6 \times 10^{-7}\ m^2\ V^{-1}\ s^{-1}$ and $\sigma_{EDL} = 0.1\ C\ m^{-2}$, we get $C_{p,1} = 0.1\ S\ m^{-1}$ and $C_{p,2} = 42.4\ S\ m^{-1}$. Thus, we obtain $\omega^2 = 3.9 \times 10^9\ s^{-2}$, $\tau_{p,1}^2 = 4.2 \times 10^{-20}\ s^2$, $\tau_{p,2}^2 = 1.1 \times 10^{-15}\ s^2$, $\omega^2\tau_{p,1}^2 \ll 1$, and $\omega^2\tau_{p,2}^2 \ll 1$. As a result, Eqs. S20 and S22 are reformulated to Eq. S27.

$$\alpha_{p,k} \approx \alpha_{p,k}^{0} = \frac{\pi\langle H\rangle\langle D\rangle^2}{6}\varepsilon_0\varepsilon_s \frac{C_{p,k}-C_s}{C_s+(C_{p,k}-C_s)\frac{F_{p,k}}{4\pi}} \quad \text{(Eq. S27)}$$

Considering the huge geometrical anisotropy of 2D VMT (where geometrical anisotropy factor $\gamma = \frac{\langle D\rangle}{\langle H\rangle}$ for 2D materials) and the conductivity difference between the EDLs of the 2D VMT and the solvent, namely $\frac{F_{p,1}}{4\pi} \approx 1 - \frac{\pi\langle H\rangle}{2\langle D\rangle} \to 1$, $\frac{F_{p,2}}{4\pi} \approx \frac{\pi\langle H\rangle}{4\langle D\rangle}$,

and $C_p \gg C_s = 5 \times 10^{-5}\, S\, m^{-1}$, Eq. S27 is further simplified to Eqs. S28 and S29.

$$\alpha_{p,1} = \frac{\pi \langle H \rangle \langle D \rangle^2}{6} \varepsilon_0 \varepsilon_s \quad \text{(Eq. S28)}$$

$$\alpha_{p,2} = \frac{\pi \langle H \rangle \langle D \rangle^2}{6} \varepsilon_0 \varepsilon_s \frac{4 \langle D \rangle}{\pi \langle H \rangle} = \frac{2 \langle D \rangle^3}{3} \varepsilon_0 \varepsilon_s \quad \text{(Eq. S29)}$$

Hence, Eq. S19 is finally written as Eq. S30 and $\Delta\alpha$ of 2D VMT liquid crystalline dispersion is thus calculated to be $-8.1 \times 10^{-27}\, F\, m^2$. Similarly, the negative sign indicates an in-plane easy axis for excess electrical polarizability, where 2D VMT tends to align parallel to the direction of the electric field (the normal director is perpendicular to the direction of electric field). Eq. S30 is reformulated to Eq. S31, where $Z_5 = -\frac{2}{3} \varepsilon_0 \varepsilon_s \langle H \rangle^3$.

$$\Delta\alpha \approx -\alpha_{p,2} = -\frac{2 \langle D \rangle^3}{3} \varepsilon_0 \varepsilon_s \quad \text{(Eq. S30)}$$

$$|\Delta\alpha| = Z_5 \gamma^3 \quad \text{(Eq. S31)}$$

**Text S10 | Experimental determination of the inherent dipole $\mu$ of 2D VMT liquid crystalline dispersion from polarization tests.**

We assume that the intrinsic polarization of $P = 0.03\, \mu C\, cm^{-2}$ in Fig. S20 originates from the $\mu$ of all the 2D VMT in the filtrated laminate film. Hence, we obtain

$$|\mu| = \frac{P V_{membrane}}{N_{2D}} = \frac{P \cdot d_{membrane} \cdot S_{membrane} \cdot \rho V_{2D}}{m_{membrane}} \quad \text{(Eq. S32)},$$

where $V$, $d$, $S$ and $m$ are respectively the volume, thickness, surface area, and mass of the filtrated laminate film, $\rho$ the density of VMT, and $V_{2D}$ the estimated volume of each 2D VMT based on $V_{2D} = \frac{\pi \langle H \rangle \langle D \rangle^2}{6}$. Following the experimental results in Fig. 2a-b, and Fig. S19, we use $\langle D \rangle = 2.6\, \mu m$, $\langle H \rangle = 1.7\, nm$, $d_{membrane} = 40\, \mu m$, $S_{membrane} = 5.0 \times 10^{-3}\, m^2$, $m_{membrane} = 50\, mg$ and $\rho = 2.5\, g\, cm^{-3}$ to estimate $\mu$. The result is finally given as $|\mu| = 1.8 \times 10^{-23}\, C\, m$.

Additionally, $\mu$ indicates the inherent electric dipole of each LC unit and also depends on the geometrical properties, which has been represented by $V_{2D}$ in Eq. S21. Combing the parameters of filtrated laminate film as a constant

$Z_4 = \rho \frac{d_{membrane} \cdot S_{membrane}}{m_{membrane}}$, we find that $|\mu| = Z_4 P V_{2D}$. For materials in the 2D limit, $\langle H \rangle$ is assumed to be a constant of around 1~2 nm. In comparison, $\langle D \rangle$ varies greatly and determines $\gamma$. Thus, we give Eq. S33 for 2D VMT liquid crystalline dispersion, which shows a positive relationship of $|\mu|$ to $P$ and $\gamma$.

$$|\mu| = 2.6 \times 10^{-27} Z_4 P \gamma^2 \quad \text{(Eq. S33)}$$

**Text S11 | Giant Kerr effect enabled by the geometrical anisotropy factor $\boldsymbol{\gamma}$ and intrinsic polarization $\boldsymbol{P}$.**

Summarizing the derivations made in Text S2, Text S8 to Text S10, we combine Eqs. S9, S18, S31, and S33 to give Eq. S34, which is valid for $\gamma \gg 1$ and 2D materials with a determined height.

$$K = Z_1 Z_2 \Delta g (\Delta\alpha + Z_2 \mu^2) \quad \text{(Eq. S9)}$$

$$|\Delta g| \approx -2.4 Z_3 \gamma^{-1} + Z_3 \quad \text{(Eq. S18)}$$

$$|\Delta\alpha| = Z_5 \gamma^3 \quad \text{(Eq. S31)}$$

$$|\mu| = 2.6 \times 10^{-27} Z_4 P \gamma^2 \quad \text{(Eq. S33)}$$

$$K = Z_1 Z_2 (-2.4 Z_3 \gamma^{-1} + Z_3)[Z_5 \gamma^3 + Z_2 (2.6 \times 10^{-27} Z_4 P \gamma^2)^2] \quad \text{(Eq. S34)}$$

Eq. S34 shows that, all the three critical parameters ($|\Delta g|$, $|\mu|$ and $|\Delta\alpha|$) that determine $K$ monotonically increase with increasing geometrical anisotropy factor of the LC units, if the dielectric properties of LC unit and dispersant of a lyotropic system are previously considered. For 2D VMT liquid crystalline dispersion with a large $\gamma > 1500$ and a $P$ of 0.03 μC cm$^{-2}$, our analyses show that $|\mu|$ dominates Eq. S9 in that $|\Delta\alpha|$ is up to $9.3 \times 10^{-27}\ F\,m^2$ and $Z_2 \mu^2$ is about $7.8 \times 10^{-26}\ F\,m^2$. Hence, Eq. S9 becomes $K = Z_1 Z_2 \Delta g (\Delta\alpha + Z_2 \mu^2) \approx Z_1 {Z_2}^2 \Delta g \mu^2$, and the Eq. S34 becomes Eq. S35.

$$K = 6.8 \times 10^{-54} Z_1 (Z_2 Z_4)^2 P^2 \gamma^4 (-2.4 Z_3 \gamma^{-1} + Z_3) \quad \text{(Eq. S35)}$$

Furthermore, $|\Delta g|$ cannot exceed the theoretical upper limit $Z_3$, thus Eq. S35 is generalized and the result gives the following, where $Z_0 = 6.8 \times 10^{-54} Z_1 Z_3 (Z_2 Z_4)^2$

$$K = Z_0 P^2 \gamma^4 \quad \text{(Eq. S36)}$$

**Text S12 | Estimation of the energy consumption of 2D VMT liquid crystalline dispersion devices.**

Generally, a 2D VMT liquid crystalline dispersion device array is composed of four parts, including a chip, relay arrays, LC pixels, and a white backlight (if needed), where the latter two mainly contribute to the energy consumption. For LC pixels, we choose the voltage and the current at the maximum contrast to examine the energy consumption, where the working voltage and current are measured to be 12.4 V and 37.3 mA. The power is thus determined to be 0.46 W. For a proof-of-concept displayable billboard with an effective area of 37.5 $cm^2$, we estimate that the power consumption (power density) is 122.7 W $m^{-2}$ for the LC pixels. In addition, there is the power of the backlight with the same area is 0.25 W, which corresponds to a consumption of 65.9 W $m^{-2}$. Thus, the power consumption of the array whose backlight is supplied from the reflection of a solar beam is approximately 123 W $m^{-2}$, and that of the array whose backlight is supplied from the light emitting diode screen is approximately 189 W $m^{-2}$. We therefore estimate the power consumption of our device arrays to be 100~200 W $m^{-2}$. Note that previous research classified display technologies with an energy consumption below 100 W $m^{-2}$ as ultra-low power-consuming [30], where the consumption of backlight is also included. Moreover, as an example, the brightness of a 2D VMT liquid crystalline dispersion device with 1) an ITO coated (or flexible) substrate, 2) an effective 2D VMT thickness of ~30 nm 3) a transparency of >85%, and 4) working in an outdoor environment, is about 300 cd $m^{-2}$ for blue light at a working current density of 2.5 mA $cm^{-2}$, which accounts for the current efficiency of 12 cd $A^{-1}$. Our device consequently outperforms other commercial outdoor candidates in terms of energy consumption or efficiency, for example, an organic LC based display or a light emitting diode display.

**Text S13 | Transient test and response time of 2D VMT liquid crystalline dispersion devices.**

Previous work shows that the transient electric birefringence test is applicable to examine the dynamic behavior and response time for a liquid crystalline dispersion

[31, 32], following

$$\Delta n_{rising}(t) = \Delta n_s \left(1 + \frac{\beta-2}{2(\beta+1)} e^{-\frac{t}{\tau_{rising}}} - \frac{3\beta}{2(\beta+1)} e^{-\frac{t}{3\tau_{rising}}}\right) + Z_5, \text{ and}$$

$$\Delta n_{falling}(t) = \Delta n_s e^{-\frac{t}{\tau_{falling}}} + Z_6,$$

where $\Delta n_{rising}$ represents the birefringence at the rising edge, $\Delta n_{falling}$ represents the birefringence at the falling edge, $\Delta n_s$ is the largest birefringence in the process, $t_{rising}$ is the characteristic time of rising edge, $t_{falling}$ is the characteristic time of falling edge, $t$ is the time, as well as $Z_5$, $Z_6$ and $\beta$ are constants. Hence, the normalized birefringence $\Delta n_{rising-normalized}$ and $\Delta n_{falling-normalized}$ gives ($Z_7$ and $Z_8$ are the constants)

$$\Delta n_{rising-normalized}(t) = 1 + \frac{\beta-2}{2(\beta+1)} e^{-\frac{t}{t_{rising}}} - \frac{3\beta}{2(\beta+1)} e^{-\frac{t}{3t_{rising}}} + Z_7$$

$$\Delta n_{falling-normalized}(t) = e^{-\frac{t}{t_{falling}}} + Z_8$$

Considering the polydispersity of the nanomaterials, a polydispersitity factor $\alpha_p$ is added [33], which gives

$$\Delta n_{rising-normalized}(t) = 1 + \frac{\beta-2}{2(\beta+1)} e^{-\left(\frac{t}{t_{rising}}\right)^{\alpha_p}} - \frac{3\beta}{2(\beta+1)} e^{-\left(\frac{t}{3t_{rising}}\right)^{\alpha_p}} + Z_7$$

$$\Delta n_{falling-normalized}(t) = e^{-\left(\frac{t}{t_{falling}}\right)^{\alpha_p}} + Z_8$$

For the data provided in Table S5 that describes the response time of the display devices, 2D VMT liquid crystalline dispersion with a volume fraction of 0.04 vol% and an electric field of $4.0\times10^3$ V m$^{-1}$ (10 kHz) are applied, thus the response time is examined with the same parameters. Fig. S32 shows that the fitting curve agrees well with our experimental results, where the $t_{rising}$ is determined to be 0.35 s and $t_{falling}$ is determined to be 2.05 s. The 0.35 s refers to the rising-time constant $t_R$ that we summarized in Table S5. Similarly, a time-dependent transmittance spectrum follows the same exponential processes, as

$$T_{rising}(t) = T_s \left(1 + \frac{\beta-2}{2(\beta+1)} e^{-\left(\frac{t}{t_{rising}}\right)^{\alpha_p}} - \frac{3\beta}{2(\beta+1)} e^{-\left(\frac{t}{3t_{rising}}\right)^{\alpha_p}}\right) + Z_9, \text{ and}$$

$$T_{falling}(t) = T_s e^{-\left(\frac{t}{t_{falling}}\right)^{\alpha_p}} + Z_{10},$$

namely,

$$T_{rising-normalized}(t) = 1 + \frac{\beta-2}{2(\beta+1)} e^{-\left(\frac{t}{t_{rising}}\right)^{\alpha p}} - \frac{3\beta}{2(\beta+1)} e^{-\left(\frac{t}{3t_{rising}}\right)^{\alpha p}} + Z_{11},$$

$$T_{falling-normalized}(t) = e^{-\left(\frac{t}{t_{falling}}\right)^{\alpha p}} + Z_{12}$$

where $T_{rising}$, $T_{falling}$, $T_s$ are transmittance at the rising edge, falling edge, and the maximum value, respectively.

Actually, the response time is obviously slower than other organic LCs, whose response time is several milliseconds or even smaller [34]. Noteworthy, we find that the characteristic response time $t_R$ is also theoretically relates to the geometrical parameters of the building blocks of the liquid crystalline system with $t_R \propto D_R^{-1}$ and $D_R \propto \langle D \rangle^{-3}$ [35, 36], where $D_R$ is the rotary diffusion coefficient and $\langle D \rangle$ is the average length of 2D platelets. It indicates that the response time has an cubic dependence on the major particle dimensions following $t_R \propto \langle D \rangle^3$. In all, 2D materials have a large length and geometric anisotropy, which contribute to an extremely high electric field sensitivity (namely, giant Kerr effect), and also make it a challenge to shorten the response time at the same time. Similar to the organic LCs, the improvement strategy may include the development of new low-viscosity system and voltage control methodology [37]. Therefore, we demonstrate a large-scale outdoor displayable billboard as a prototype, where a sensitive response is relatively of more importance.

## Supplementary Figures

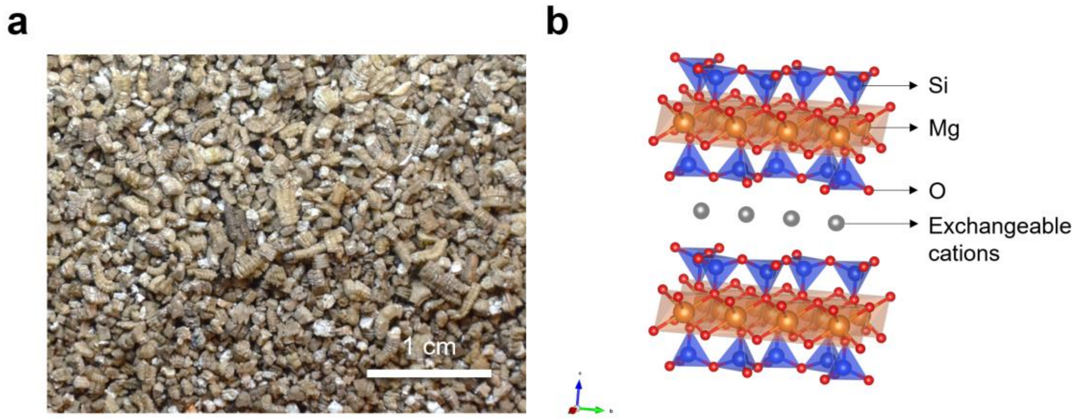


**Fig. S1 | Bulk VMT and its atomic structure.**

**a**, Optical image of bulk VMT mineral with an average size of 2-3 mm. **b**, Structure of VMT. Each layer is composed of an Mg-based octahedral sheet (in orange) sandwiched between a pair of Si-based tetrahedral sheets (in blue). Elemental substitution by Al, Fe, etc. in the sheets often occurs. Some exchangeable cations are commonly stored in gaps between the layers.

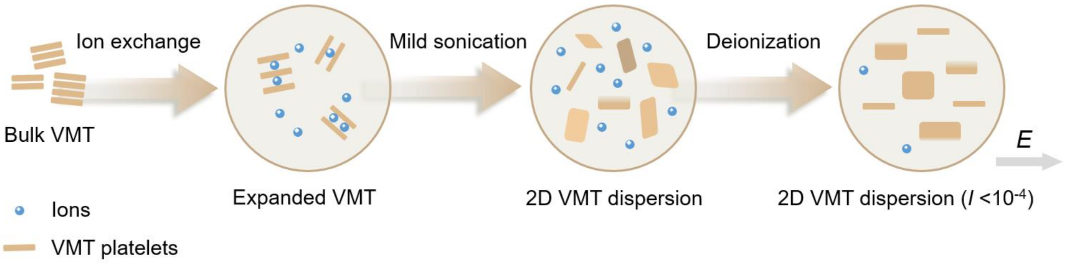


**Fig. S2 | Preparation of 2D VMT dispersion.**

A schematic showing the preparation process of 2D VMT liquid crystalline dispersion, which includes three main procedures of ion exchange, mild sonication, and deionization. The ionic strength of 2D VMT liquid crystalline dispersion is below $10^{-4}$.

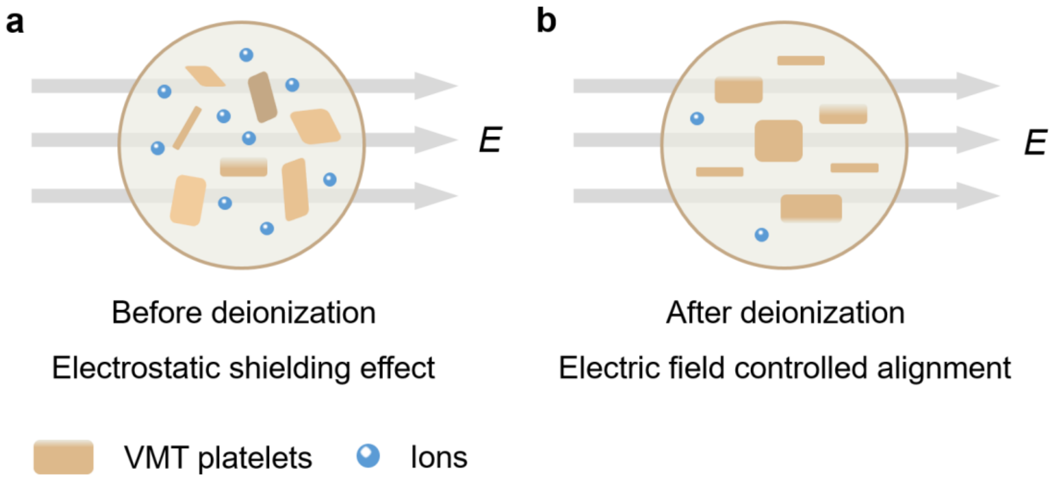


**Fig. S3 | Electrostatic shielding effect of ions.**

Schematics of 2D VMT liquid crystalline dispersion (**a**) before and (**b**) after deionization. The ions lead to electrostatic shielding effect.

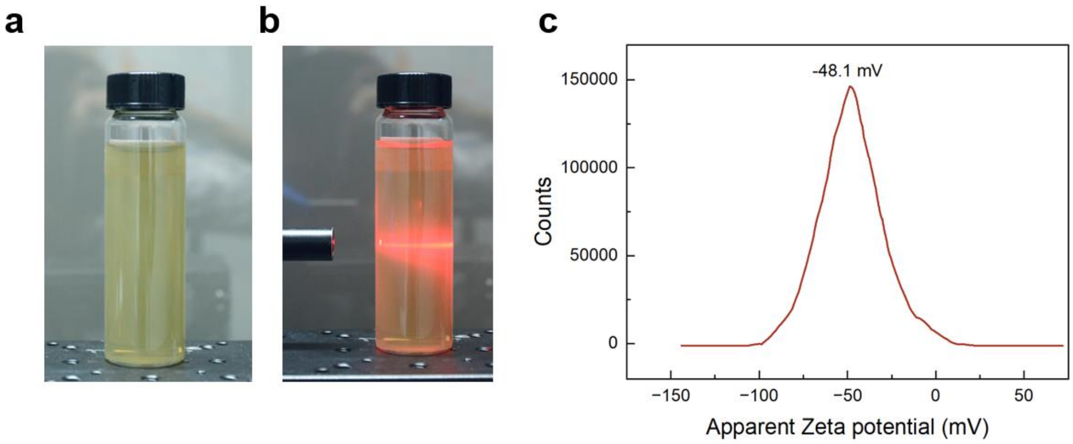


**Fig. S4 | Characterization of 2D VMT liquid crystalline dispersion.**

**a-b**, Optical images of (**a**) a 2D VMT liquid crystalline dispersion and (**b**) its Tyndall effect. **c**, Zeta potential of a 2D VMT liquid crystalline dispersion with a peak at -48.1 mV.

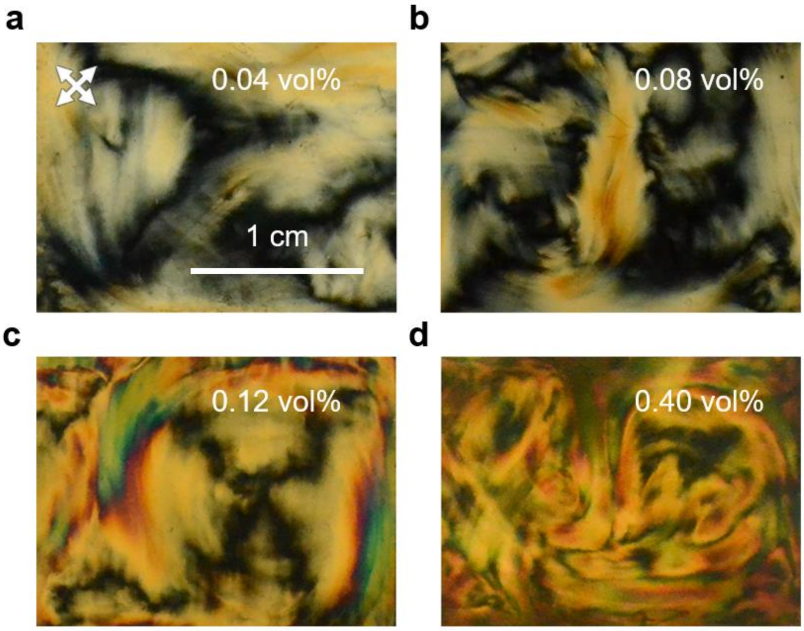


**Fig. S5 | Phase transition behavior of 2D VMT liquid crystalline dispersion.**

**a**-**c**, Flow-induced birefringent textures of 2D VMT liquid crystalline dispersion with the volume fractions of (**a**) 0.04 vol%, (**b**) 0.08 vol%, and (**c**) 0.12 vol%. More colors appear in bright fringes as the volume fraction increases. **d**, Volume-fraction-induced stable Schlieren textures of 2D VMT liquid crystalline dispersion in the nematic phase.

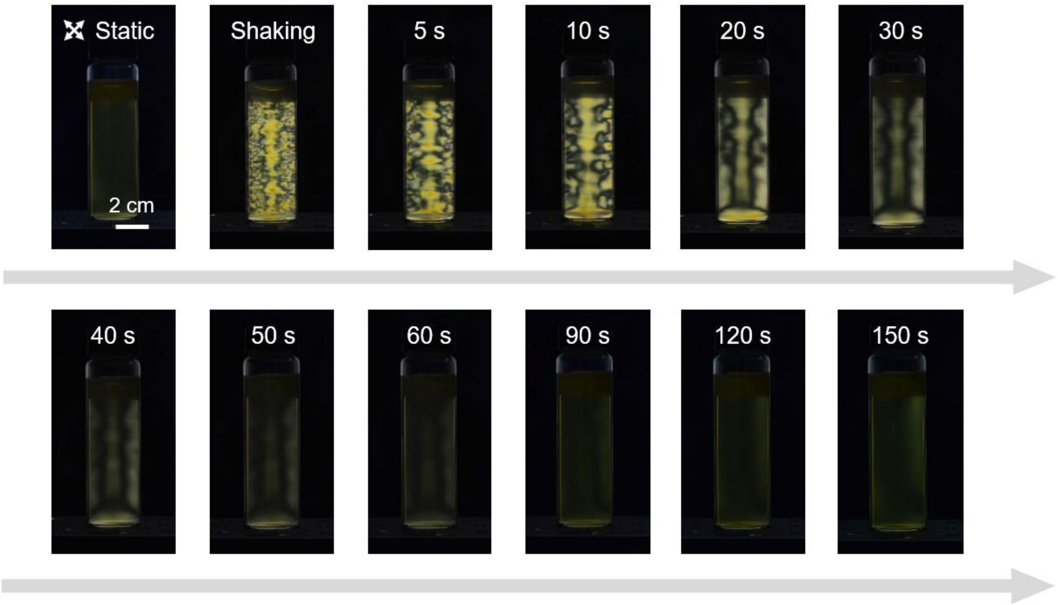


**Fig. S6 | Global observation of flow-induced birefringence of 2D VMT liquid crystalline dispersion with a volume fraction of 0.08 vol%.**

Polarized optical images of 2D VMT liquid crystalline dispersion with a volume fraction of 0.08 vol% at a steady state and after shaking of 0, 5, 10, 20, 30, 40, 50, 60, 90, 120, and 150 seconds.

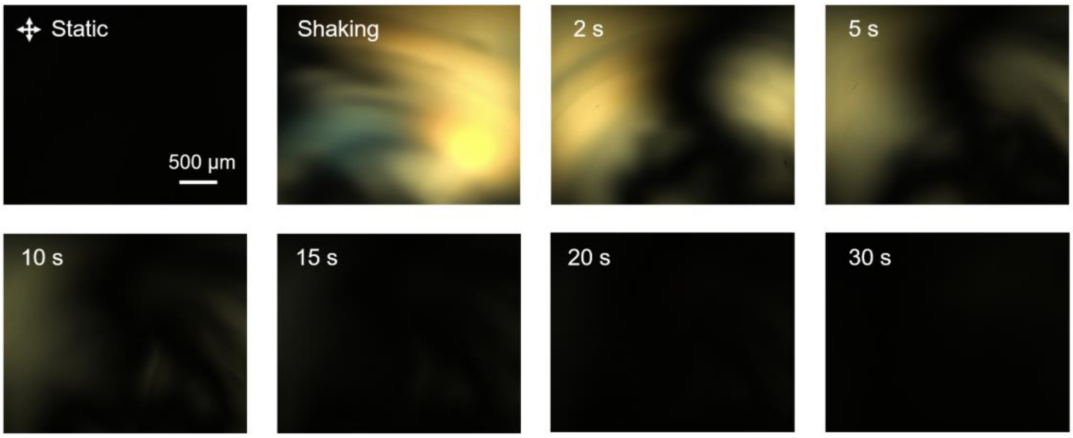


**Fig. S7 | Local observation of flow-induced birefringence of 2D VMT liquid crystalline dispersion with a volume fraction of 0.08 vol%.**

POM images of 2D VMT liquid crystalline dispersion with a volume fraction of 0.08 vol% at a static state and after shaking of 0, 2, 5, 10, 15, 20, and 30 seconds.

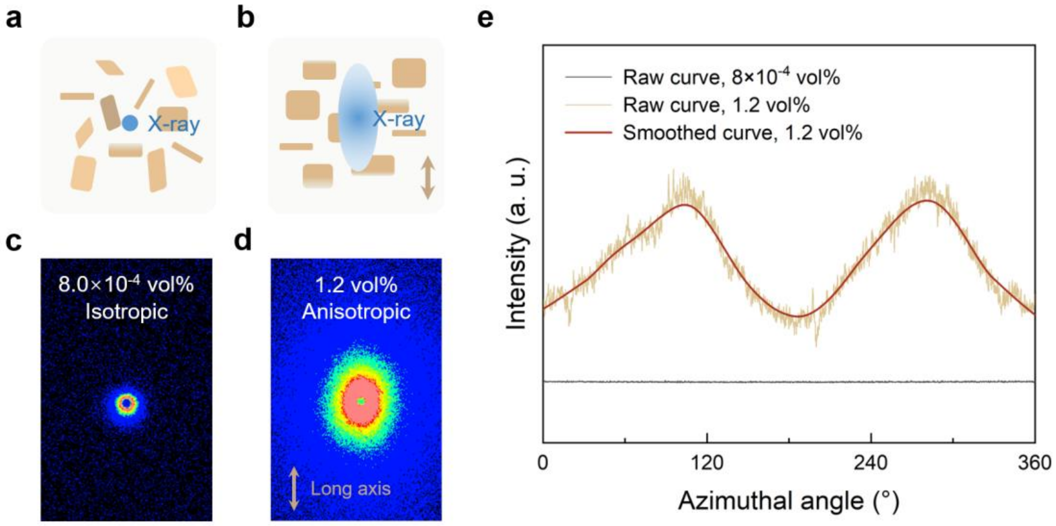


**Fig. S8 | SAXS of 2D VMT liquid crystalline dispersion with volume fractions of $8.0\times10^{-4}$ and 1.2 vol%.**

**a-b,** Schematic of a 2D VMT liquid crystalline dispersion in an optically isotropic state ($8.0\times10^{-4}$ vol%) (**a**) and in an optically anisotropic state (1.2 vol%) (**b**). **c-d,** SAXS scattering patterns of 2D VMT liquid crystalline dispersion with a volume fraction of $8.0\times10^{-4}$ vol% (**c**) and 1.2 vol% (**d**). The arrow in brown indicates the direction of long axis of the elliptical pattern. **e**, Scattering profiles. There exists no peak for the 2D VMT liquid crystalline dispersion at isotropic state with a volume fraction of $8.0\times10^{-4}$ vol%. The 2D VMT liquid crystalline dispersion with a volume fraction of 1.2 vol% shows anisotropic scattering with two emerging peaks.

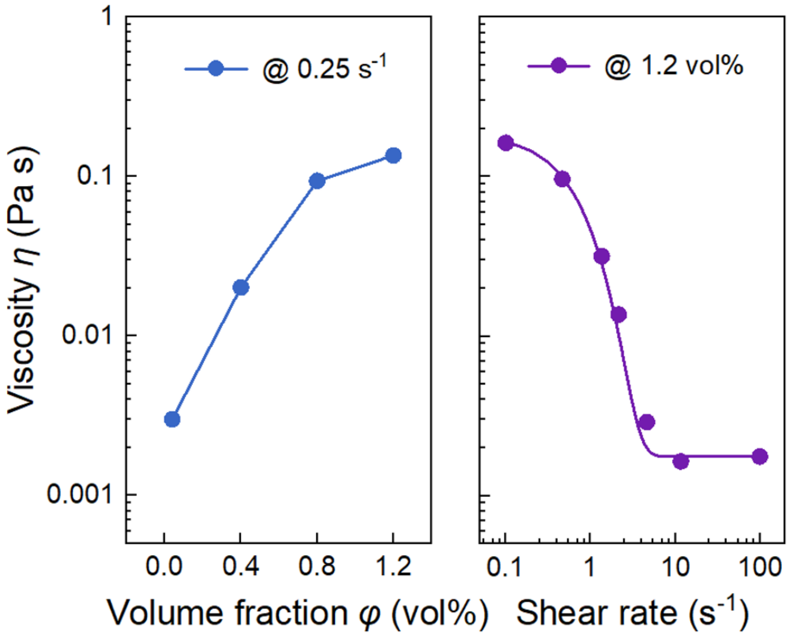


**Fig. S9 | Rheological tests of 2D VMT liquid crystalline dispersion.**

The left panel shows a volume-fraction-induced thickening behavior at a shear rate of 0.25 $s^{-1}$, where the viscosity increases as the volume fraction of 2D VMT liquid crystalline dispersion increases. The right panel shows that the viscosity of 2D VMT liquid crystalline dispersion with the volume fraction of 1.2 vol% decreases with increasing the shear rate, indicating a shear thinning effect.

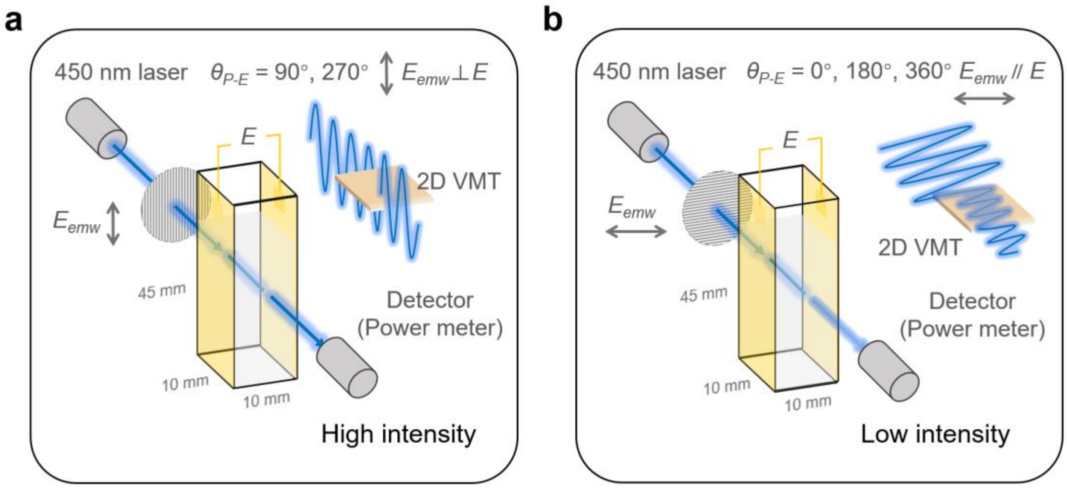


**Fig. S10 | Schematics of the polarization-dependent transmittance.**

The optical setup includes a 450 nm laser, a polarizer, a cuvette with counter electrodes and 2D VMT liquid crystalline dispersion, and a detector (power meter).

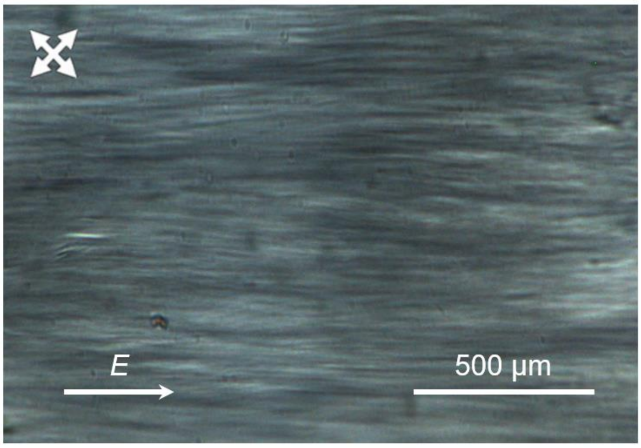


**Fig. S11 | A POM image showing parallel textures.**

The textures are parallel with the direction of the electric field ($2.0 \times 10^4$ V $m^{-1}$, 10 kHz).

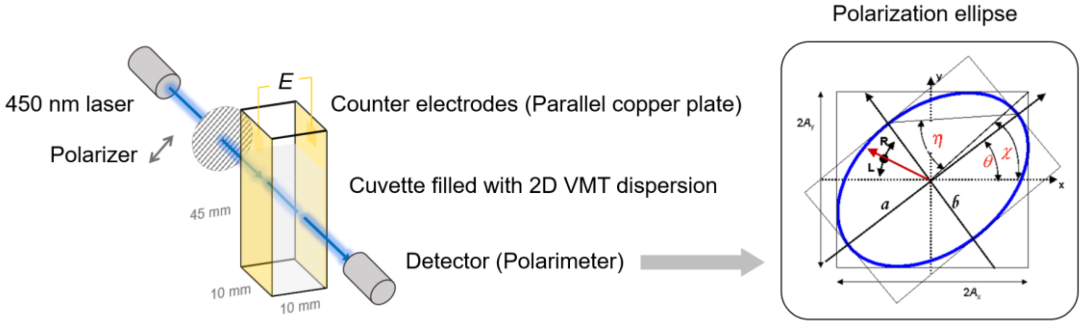


**Fig. S12 | Schematics of the Kerr experiments.**

The optical setup includes a 450 nm laser, a polarizer (the polarization direction 45 degrees to the direction of electric field), a cuvette with counter electrodes and 2D VMT liquid crystalline dispersion, and a detector (polarimeter). The polarimeter outputs a polarization ellipse, where the azimuth angle and the ellipticity are used to calculate the birefringence and the Kerr coefficient.

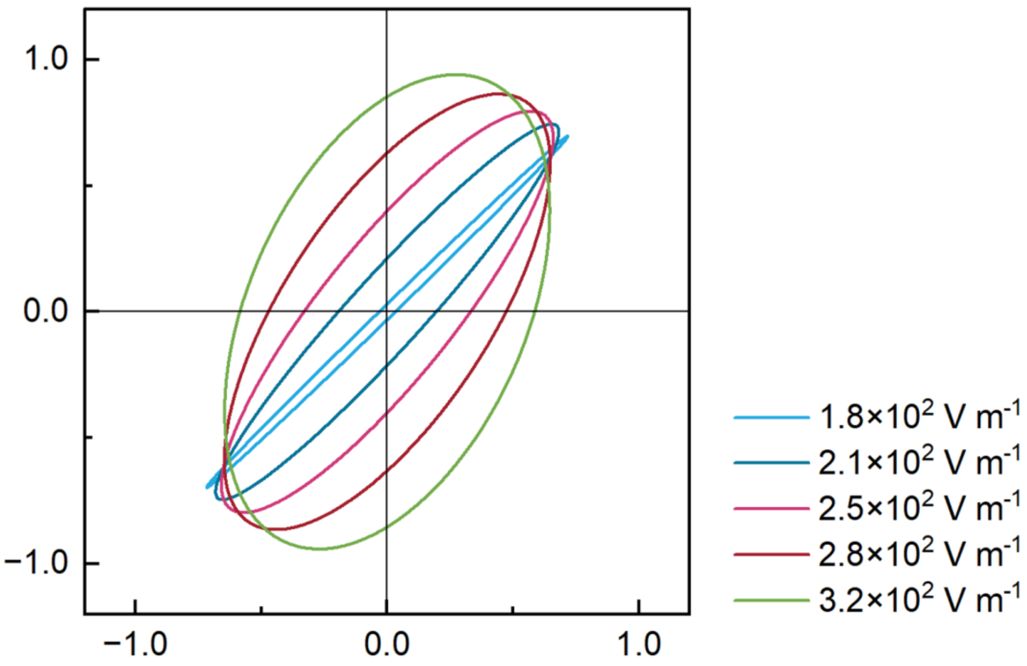


**Fig. S13 | Polarization ellipse of 2D VMT liquid crystalline dispersion with a volume fraction of 0.08 vol%.**

The electric field is set to be $1.8\times10^2$, $2.1\times10^2$, $2.5\times10^2$, $2.8\times10^2$, and $3.2\times10^2$ V $m^{-1}$ (10 kHz).

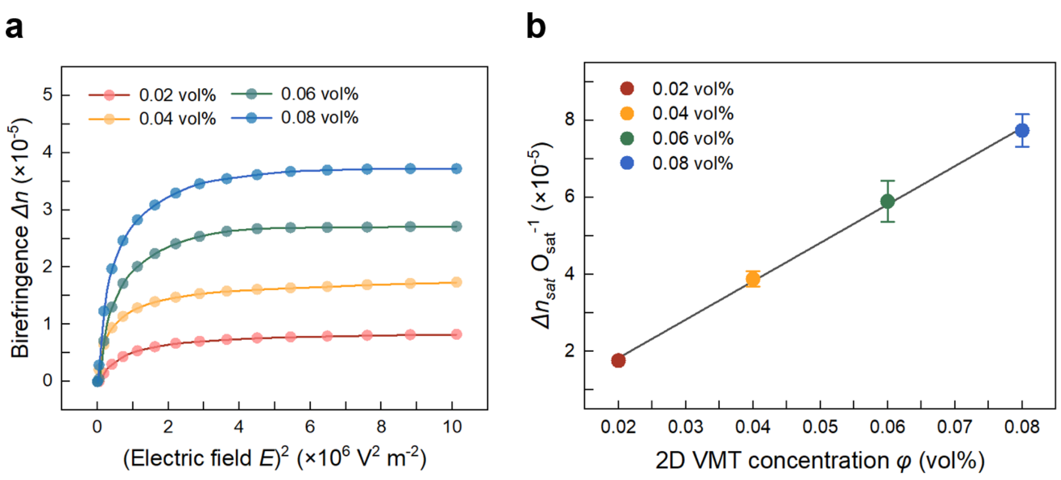


**Fig. S14 | Saturated birefringence of 2D VMT liquid crystalline dispersion.**

**a**, Electro-birefringence for 2D VMT liquid crystalline dispersion with volume fractions of 0.02, 0.04, 0.06 and 0.08 vol% in the electric field range of 0 to $3.2\times10^{3}$ V $m^{-1}$ (10 kHz). **b**, Linear fit between saturated birefringence and volume fraction.

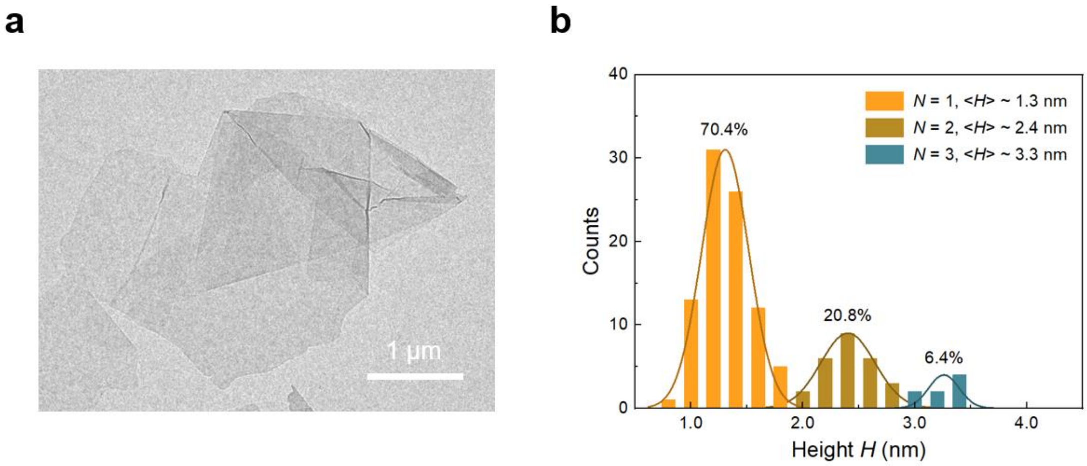


**Fig. S15 | Morphology characterization on 2D VMT liquid crystalline dispersion.** **a**, TEM image of 2D VMT dropped onto a copper mesh. **b**, Statistics for the height $H$ of 2D VMT, counted from AFM images.

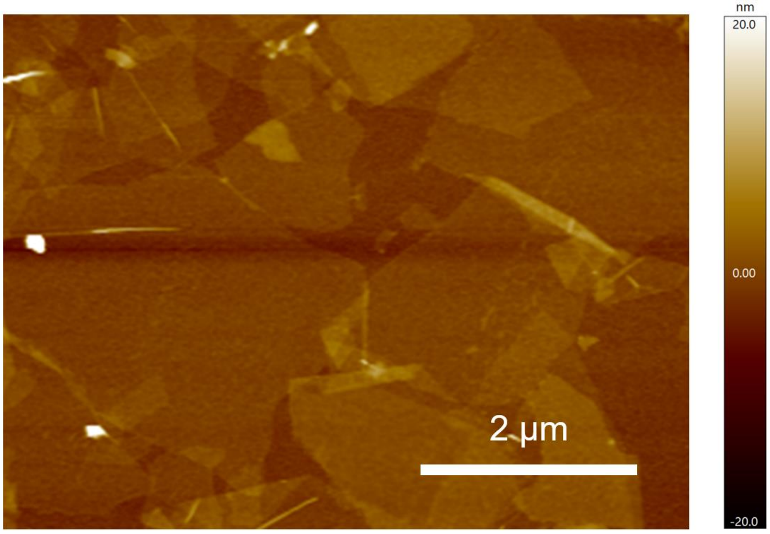


**Fig. S16 | 2D VMT coated on a conducting Au substrate for PFM measurement.** 2D VMT is transferred from a dispersion to an Au substrate by the Langmuir−Blodgett method with a surface pressure of 10 mN $m^{-1}$.

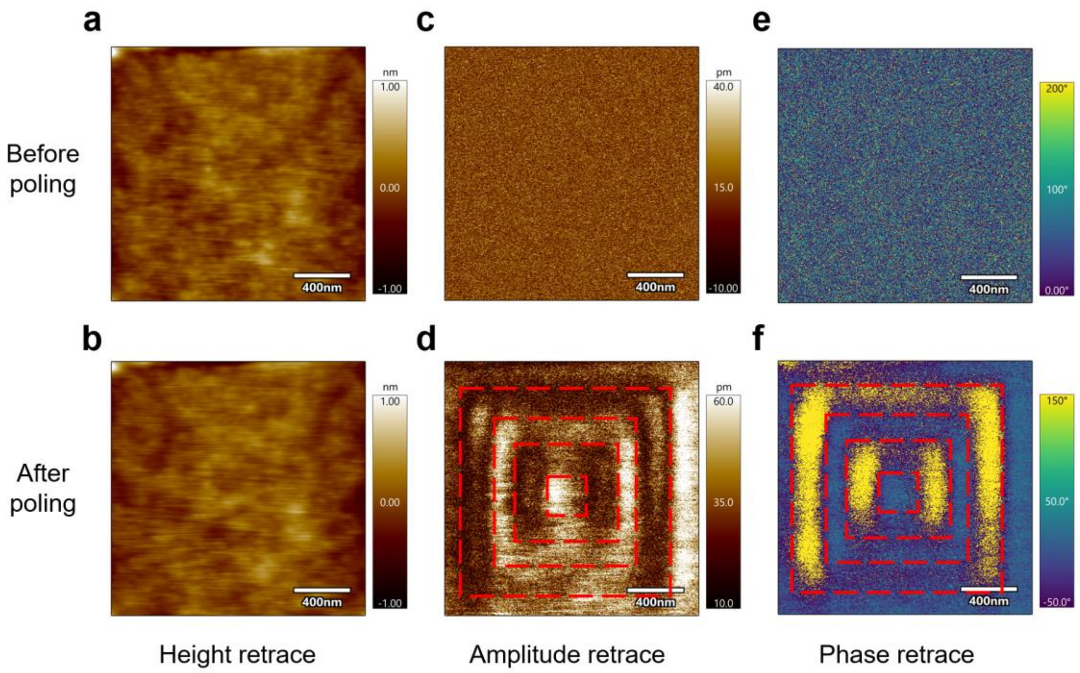


**Fig. S17 | Polarization switching of 2D VMT by PFM.**

**a**, **c**, and **e**, Background images of (**a**) height, (**c**) amplitude, and (**e**) phase retrace before poling. **b**, **d**, and **f**, Maps of (**b**) height, (**d**) amplitude, and (**f**) phase retrace after applying a reverse DC bias. The written box-in-box patterns are seen in both the amplitude and phase retrace with no change on its morphology.

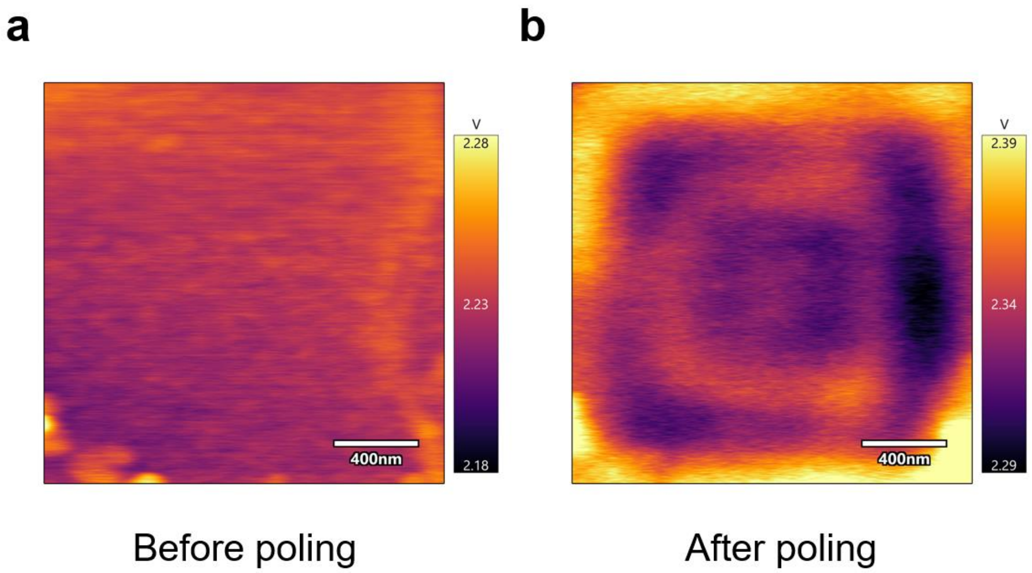


**Fig. S18 | Surface potential of 2D VMT by KPFM.**

**a**, Background image. **b**, Map of surface potential after poling. The potential difference is over 100 mV.

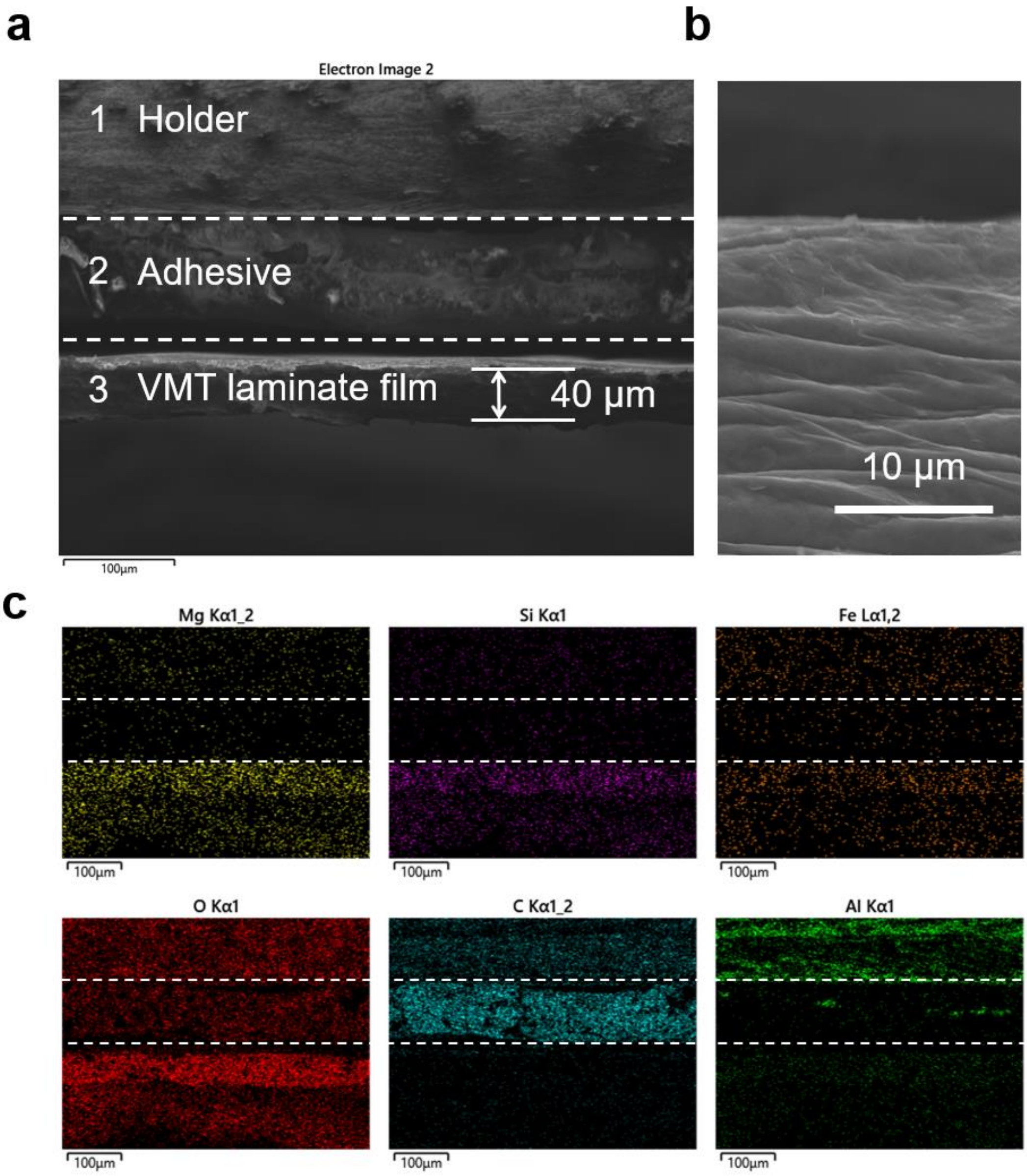


**Fig. S19 | 2D VMT laminate film used for the polarization test.**

**a**, SEM image of the 2D VMT laminate film with a thickness of approximately 40 μm. **b**, A zoom-in image showing layered stacking in the film. **c**, EDX images.

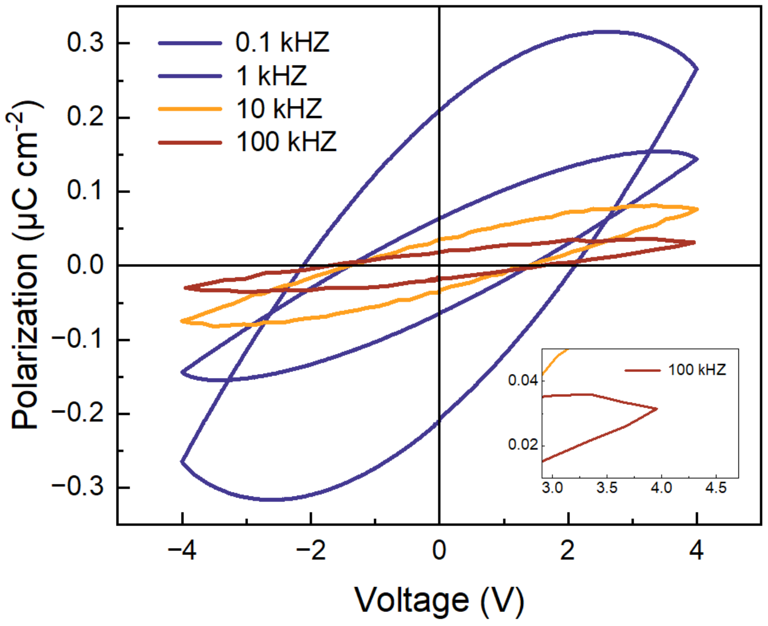


**Fig. S20 | Polarization *versus* voltage hysteresis loops of an assembled 2D VMT laminate film at different frequencies.**

The maximum voltages are -4 V and 4 V. The residual polarization is approximately 0.03 μC $cm^{-2}$ at a frequency of 100 kHz.

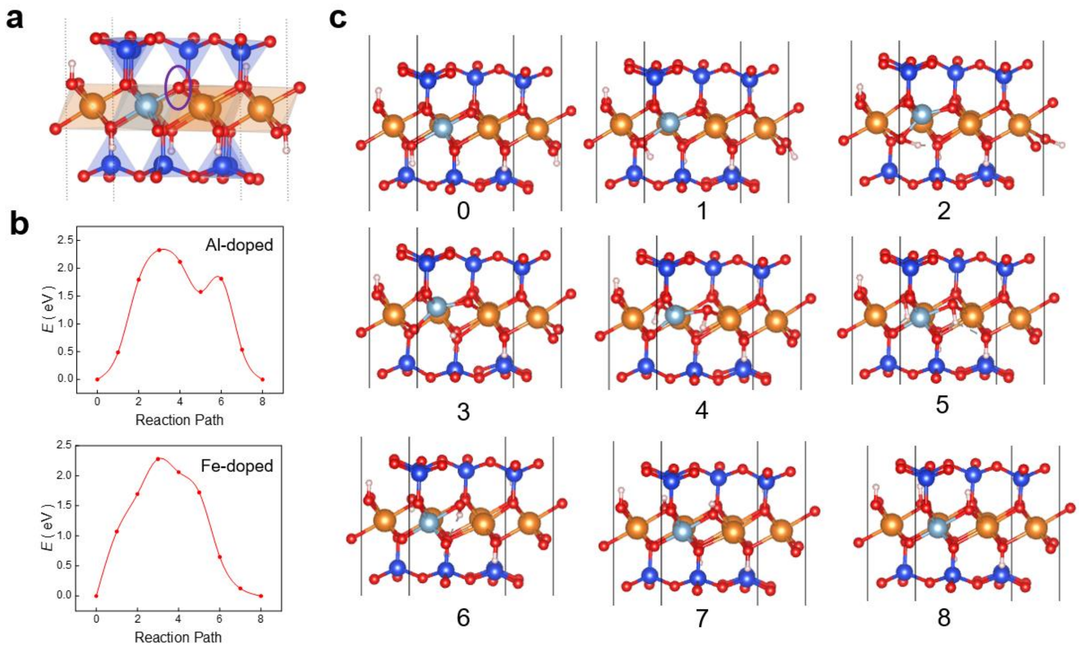


**Fig. S21 | Crystalline structure of 2D VMT and the origin of ferroelectricity.**

**a**, The structure of a monolayer vermiculite $(Mg_6Si_8O_{20})(OH)_4$ with a proton vacancy marked in a circle. **b**, Ferroelectric switching pathway for Al or Fe doped $(Mg_6Si_8O_{20})(OH)_4$ monolayer. **c**, the snapshots during switching.

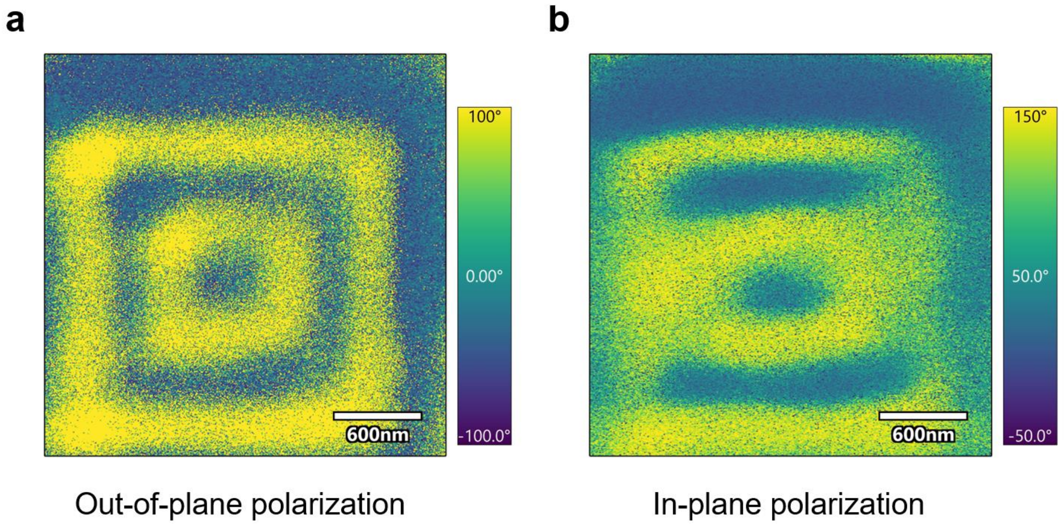


**Fig. S22 | PFM phase maps of 2D VMT.**

**a,** A map showing a flipped polarization in the out-of-plane direction. **b**, A map showing a flipped polarization in the in-plane direction.

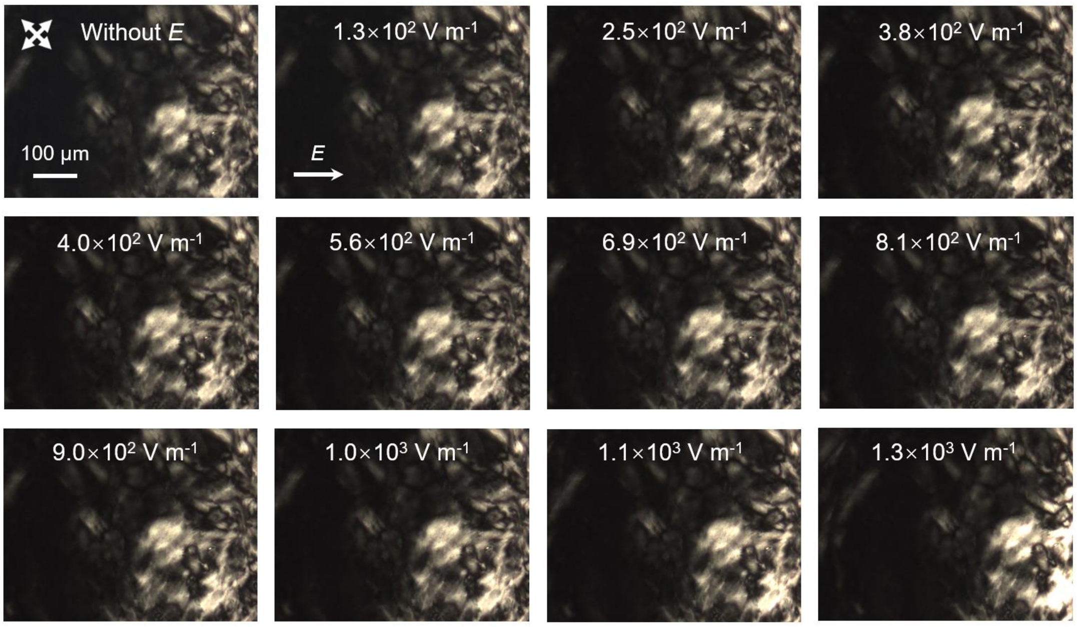


**Fig. S23 | POM images of 2D VMT dispersion with a volume fraction of 2.0 vol%.**

A DC electric field with the strength from 0 to $1.3\times10^{3}$ V m$^{-1}$ is applied. A higher DC electric field leads to electrophoresis.

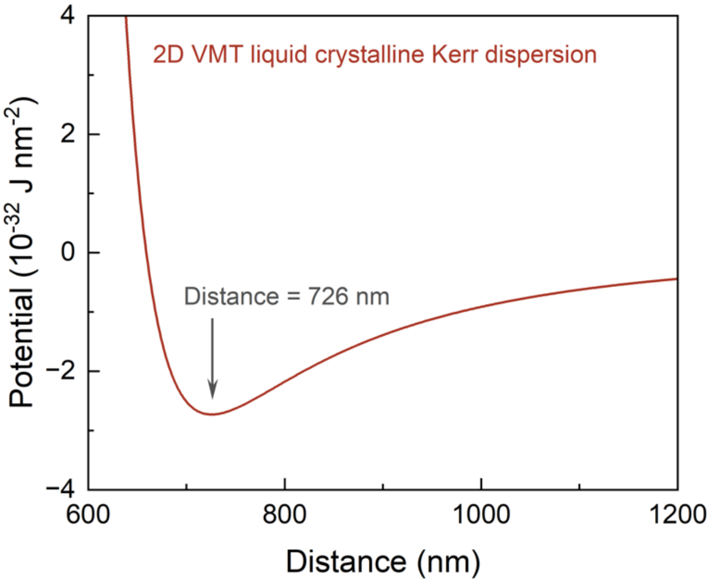


**Fig. S24 | Theoretical interaction potential between 2D VMT platelets.**

The potential curve is calculated following the DLVO theory. The ionic strength of 2D VMT liquid crystalline dispersion is $1\times10^{-4}$.

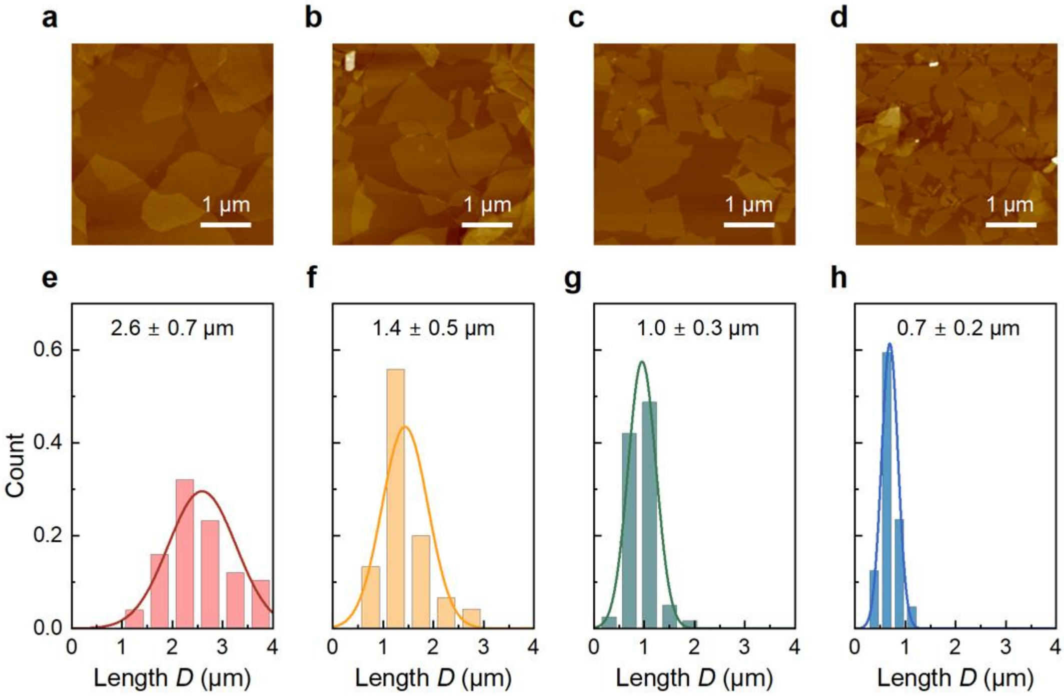


**Fig. S25 | AFM images and statistics of 2D VMT liquid crystalline dispersion in which 2D VMT has different lengths.**

**a-d**, AFM images of 2D VMT, where its dispersion is sonicated by 0 (**a**), 30 (**b**), 60 (**c**), and 120 (**d**) minutes. **e-g**, Statistics showing that the average lengths of 2D VMT in (**a-d**) are approximately 2.6 (**e**), 1.4 (**f**), 1.0 (**g**), and 0.7 (**h**) μm.

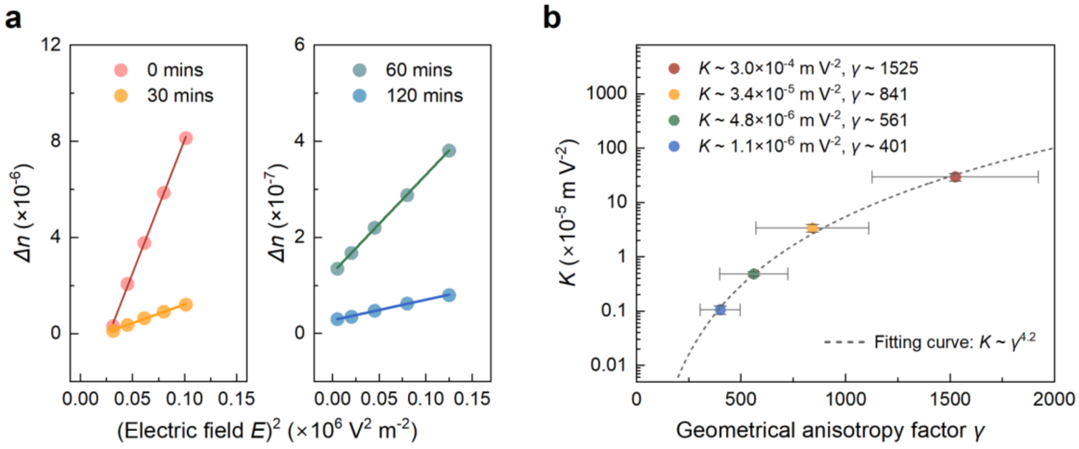


**Fig. S26 | Kerr coefficients of 2D VMT liquid crystalline dispersion in which 2D VMT has different geometrical anisotropy factor.**

**a**, Kerr coefficient of 2D VMT dispersion that is sonicated by 0, 30, 60, and 120 minutes. **b**, Dependence of Kerr coefficient with geometrical anisotropy factor. The Kerr coefficient is determined to be $3.0\times10^{-4}$, $3.4\times10^{-5}$ m $V^{-2}$, $4.8\times10^{-6}$ m $V^{-2}$, and $1.1\times10^{-6}$ m $V^{-2}$, meeting a fitting curve of $K \propto \gamma^{4.2}$.

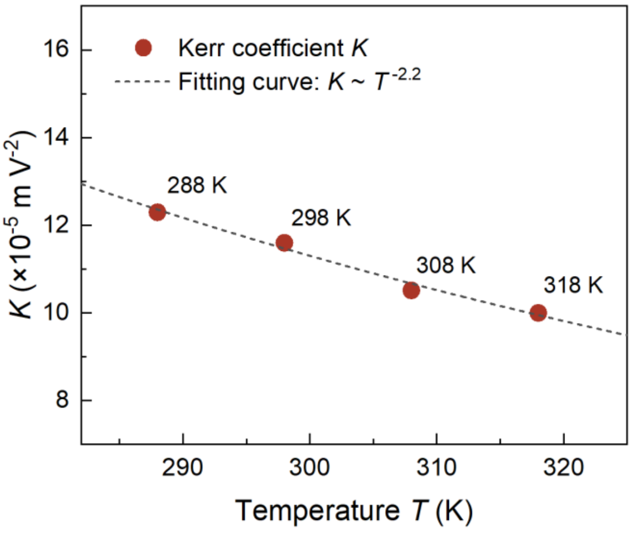


**Fig. S27 | Dependence of the Kerr coefficients with temperature.**

Kerr coefficients of 2D VMT dispersion with a volume fraction of 0.04 vol%. The electric field is in 10 kHz. The fitting curve gives a relationship of $K \propto T^{-2.2}$.

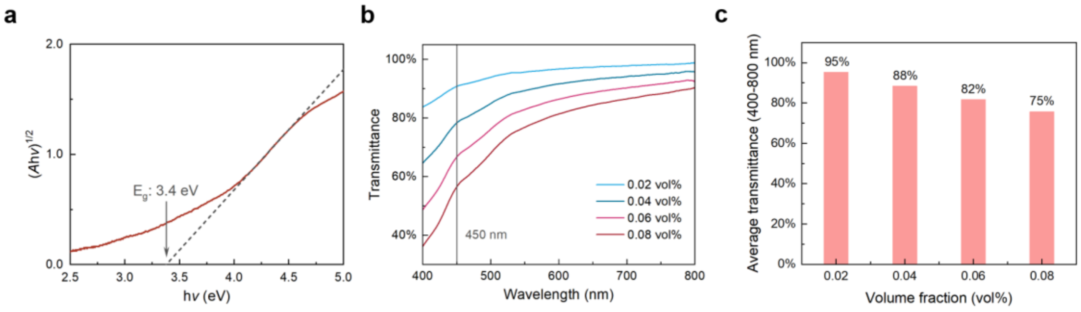


**Fig. S28 | Optical band gap of 2D VMT liquid crystalline dispersion and its transmittance.**

**a**, Tauc plot. *A* is the absorbance and h*ν* is the photon energy. **b**, Transmittance of 2D VMT liquid crystalline dispersion with volume fractions of 0.02, 0.04, 0.06, and 0.08 vol%. **c**, Average transmittance of them for all the visible light (400-800 nm).

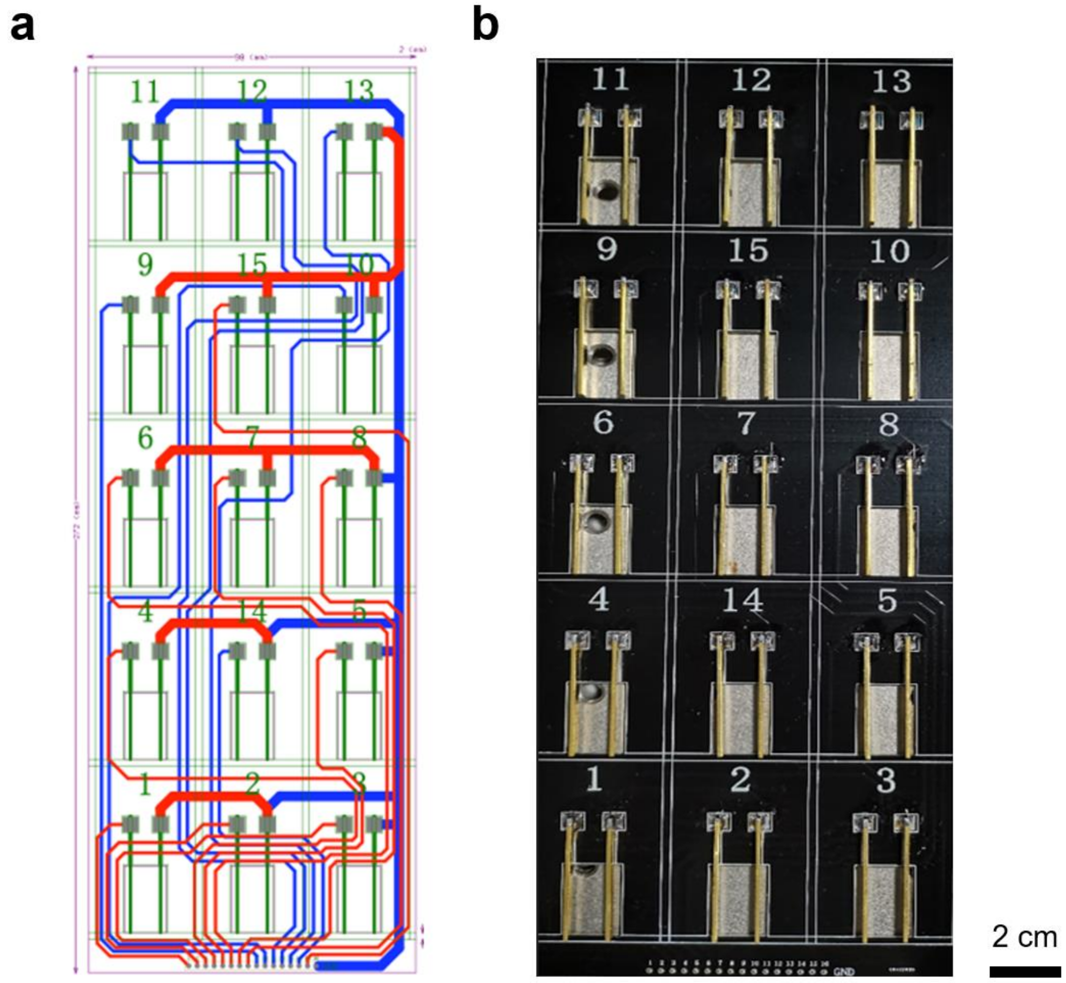


**Fig. S29 | Custom-made device array integrating 15 displayable macroscopic pixels.**

**a**, Design diagram. **b**, Optical image of the device array produced on a printed circuit board. Copper plate electrodes are welded with a separation of 10 mm. Cuvettes with 2D VMT liquid crystalline dispersion are loaded as the pixels.

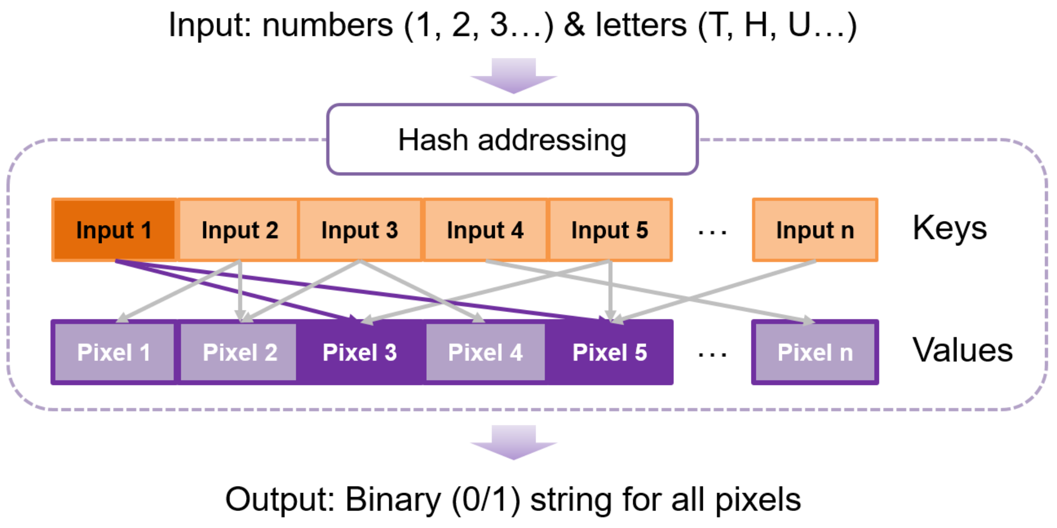


**Fig. S30 | A mechanism schematic of the hash addressing algorithm.**

The Arduino chip receives an input signal. The hash addressing algorithm outputs a binary 15-digit string. Each digit in this string refers to the state of a pixel.

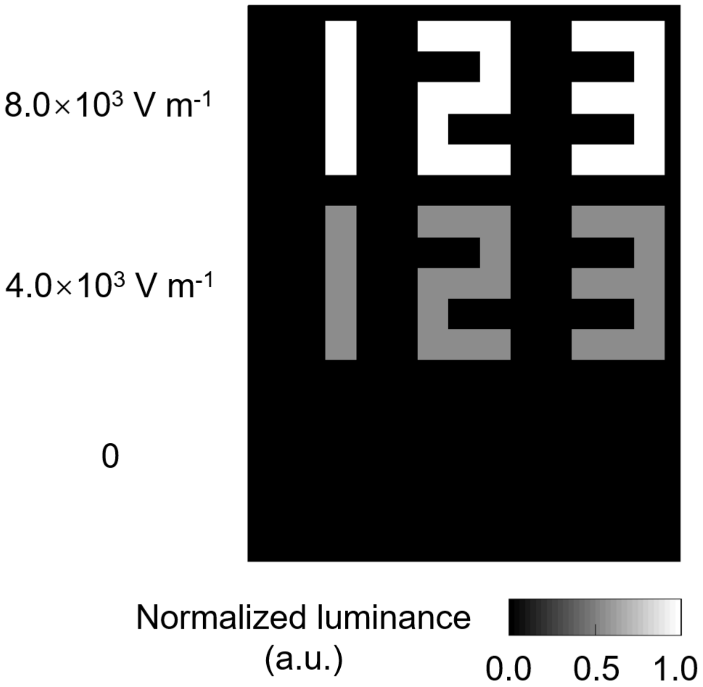


**Fig. S31 | Luminance test on the pixels of the gesture capture array.**

The electric field is preset to $4.0\times10^3$ V m$^{-1}$ or $8.0\times10^3$ V m$^{-1}$. The output value comes out as binary 15-digit strings of '001010010100100', '111101110111100', and '111011110111100' for input signals of numbers of '1', '2', and '3', where "1" in these strings indicate that the relays that control the pixels with corresponding notes in Fig. S29 will be turned on. The map shows a display uniformity of 97% among all pixels at each state.

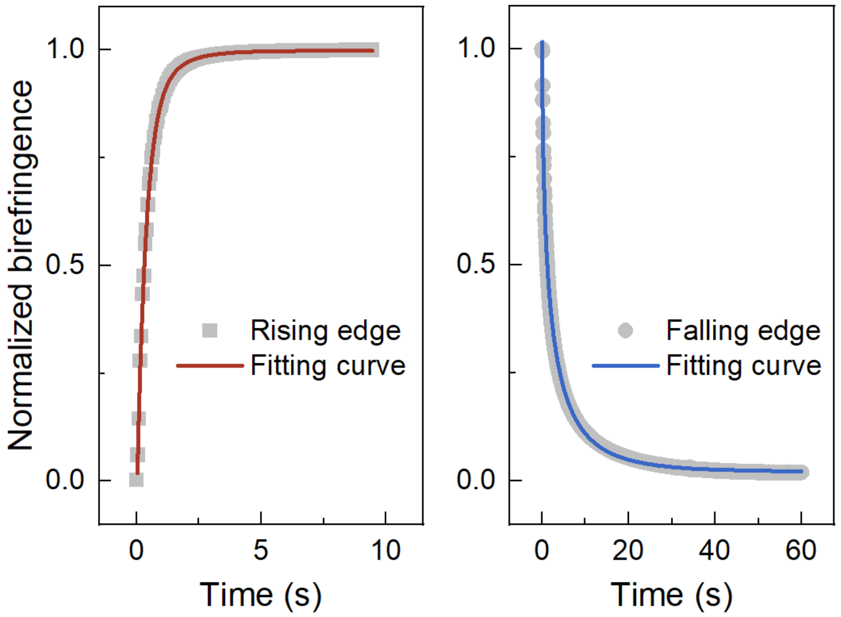


**Fig. S32 | Transient electric birefringence test of 2D VMT liquid crystalline dispersion device.**

The left panel refers to a rising edge and the right panel refers to a falling edge. The electric field is set to be $4.0\times10^{3}$ V $m^{-1}$ (10 kHz).

**Table S1 | Azimuth angle, ellipticity, and birefringence of 2D VMT liquid crystalline dispersion with a volume fraction of 0.08 vol%.**

The data are used in Fig. 1j and Fig. S13.

| **Volume fraction (vol%)** | **Electric field ($10^2$ V m$^{-1}$)** | **Azimuth angle $\theta$ (degree)** | **Ellipticity $\eta$ (degree)** | **Birefringence** |
|---|---|---|---|---|
| | 1.8 | 44.18 | 1.28 | $3.20\times10^{-7}$ |
| | 2.1 | 47.65 | 8.25 | $2.07\times10^{-6}$ |
| 0.08 | 2.5 | 51.22 | 14.79 | $3.77\times10^{-6}$ |
| | 2.8 | 56.59 | 22.23 | $5.86\times10^{-6}$ |
| | 3.2 | 66.23 | 28.89 | $8.14\times10^{-6}$ |

**Table S2 | Comparison of the Kerr coefficient $K$ of 2D VMT liquid crystalline dispersion with other electro-optical Kerr media.**

The Kerr media are divided into four main categories, including gases, liquids, organic LCs and inorganic nanomaterials LCs, where O-LCs, O-BP-LCs, O-FE-LCs, 1D-LCs, 2D-LCs, and 2D-FE-LCs indicate classical organic LCs, organic blue phase LCs, organic ferroelectric LCs, inorganic LCs (or LC-like dispersion) based on one-dimensional materials, inorganic LCs (or LC-like dispersion) based on 2D materials, and inorganic LCs (or LC-like dispersion) based on 2D ferroelectric materials, respectively. These data are used to plot Fig. 1l.

| **Modulation media** | $\lambda$ **(nm)** | $K$ **(m V$^{-2}$)** |
|---|---|---|
| **Gases** | | |
| Oxygen [38] | 650 | $7.7\times10^{-19}$ |
| Air [38] | 633 | $2.3\times10^{-18}$ |
| Nitrogen [39] | 633 | $2.0\times10^{-18}$ |
| Carbon dioxide [39] | 633 | $5.6\times10^{-18}$ |
| Sulphur hexafluoride [39] | 633 | $8.4\times10^{-19}$ |
| Trifluoroiodomethane [39] | 633 | $4.4\times10^{-17}$ |
| **Liquids** | | |
| Water [40] | 633 | $3.0\times10^{-14}$ |
| Methanol [40] | 633 | $2.2\times10^{-14}$ |
| Acetonitrile [40] | 633 | $5.0\times10^{-13}$ |
| Chlorobenzene [40] | 633 | $1.2\times10^{-13}$ |
| Acetone [40] | 633 | $2.1\times10^{-13}$ |
| Nitromethane [40] | 633 | $1.3\times10^{-13}$ |
| Transformer oil [39] | 633 | $3.5\times10^{-15}$ |
| Nitrobenzene [39] | 546 | $4.5\times10^{-12}$ |
| m-Dinitrobenzene [41] | N/A | $1.1\times10^{-11}$ |
| **Organic LCs** | | |

| | | |
|---|---|---|
| O-LC<br>12OCB [42] | 633 | $3.3\times10^{-11}$ |
| O-LC<br>$ILC_A$ [42] | 633 | $6.4\times10^{-11}$ |
| O-LC<br>$ILC_h$ [42] | 633 | $1.5\times10^{-10}$ |
| O-LC<br>5CB [42] | 633 | $2.2\times10^{-10}$ |
| O-LC<br>9CHBT [43] | 633 | $1.1\times10^{-10}$ |
| O-LC<br>W-1680 [43] | 633 | $1.3\times10^{-10}$ |
| O-LC<br>5CB/7CB [44] | 633 | $1.5\times10^{-10}$ |
| O-BP-LC<br>JC1041/5CB/ZLI-4572 [45] | 632 | $3.7\times10^{-10}$ |
| O-BP-LC<br>JC1041/5CB/ZLI-4572 [46] | 633 | $2.0\times10^{-9}$ |
| O-BP-LC<br>JC-BP06N/5CC3 [47] | 633 | $2.7\times10^{-8}$ |
| O-BP-LC<br>JC-BP06N/5CC3 [47] | 514 | $3.3\times10^{-8}$ |
| O-BP-LC<br>JC-BP06N/5CC3 [47] | 457 | $4.0\times10^{-8}$ |
| O-FE-LC<br>FLC-587 [48] | 543 | $2.7\times10^{-8}$ |
| O-FE-LC<br>FLC-622 [49] | 450 | $1.0\times10^{-7}$ |
| O-FE-LC | 543 | $1.3\times10^{-7}$ |

| | | |
|---|---|---|
| FLC-618 [50] | | |
| **Inorganic nanomaterials LCs** | | |
| 1D-LC<br>Carbon nanotube [51] | 633 | $2.1\times10^{-11}$ |
| 1D-LC<br>Lanthanum orthophosphate [52] | 633 | $3.5\times10^{-9}$ |
| 2D-LC<br>Gibbsite [53] | 632 | $1.6\times10^{-9}$ |
| 2D-LC<br>Beidellite [15] | 546 | $1.1\times10^{-6}$ |
| 2D-LC<br>Graphene oxide [11] | 633 | $1.4\times10^{-5}$ |
| **2D-FE-LC**<br>**Vermiculite (This work)** | **450** | $\mathbf{3.0\times10^{-4}}$ |

**Table S3 | Pseudo code used for the displayable array in the Figure 3 of the main text.**

The hash addressing algorithm is written in python language and is written into the Arduino chip after compilation.

---

**Algorithm**: Novel Search for Dynamic Display

---

```
def search (mode, input_sequences):
If mode not in modes_list do
    Raise ValueError
If mode = number/letter do
    For each character in input_sequences do
        If character not in keys of number/letter map do
            Raise ValueError
        Else do
            matrix = number/letter map[character]
            For element in matrix do
                digitWrite(element, High/Low)
                time.sleep(delay_time)
            End for
    End for
```

**Table S4 | Estimation of energy consumption of the displayable array based on 2D VMT liquid crystalline dispersion.**

The power consumption of the array whose backlight is supplied from the reflection of a solar beam is approximately 123 W $m^{-2}$, and that of the array whose backlight is supplied from the light emitting diode screen is approximately 189 W $m^{-2}$.

| **Module** | **Current (mA)** | **Voltage (V)** | **Power (W)** | **Area ($cm^2$)** | **Energy Consumption (W $m^{-2}$)** |
|---|---|---|---|---|---|
| **Pixel array** | 37.2 | 12.5 | 0.46 | 37.5 | 122.7 |
| **Backlight** | - | - | 0.25 | 37.5 | 65.9 |

**Table S5 | Comparison of 2D VMT liquid crystalline dispersion devices with other electrochromic techniques.**

LC displays and electrochromic devices (ECD) based on both polymers and metal oxides are considered. Manipulating field (*E*), optical density ($\Delta OD$), response time ($t_R$), and contrast ratio (*CR*) are compared to evaluate the device performance, and the results are provided below. Note that outdoor stability (represented by life in ultraviolet irradiation, $t_{UV}$) is added considering their outdoor use. Unfortunately, it is general recognized that the outdoor performance of LC displays and polymer based ECDs are relative poor considering the organic feature of polymeric LC molecules and electrochromic polymers [54-57], so that few reports exist. For a metal oxide based ECD, a polymeric film or polymer electrolyte are also used [55, 58]. In this work, the inorganic 2D VMT liquid crystalline dispersion shows an outdoor stability with a decay of <1% after 1000 hours under sunlight. We summarize these results below and give a rating in Fig. 3g

| **Modulation media** | ***E* ($10^3$ V $m^{-1}$)** | **$\Delta OD$ (a.u.)** | **$t_R$ (s)** | ***CR* (%)** |
|---|---|---|---|---|
| LC displays [59] | >1000 | >2 | <0.01 | ~100 |
| Polymer based ECD [55] | >10 | >0.5 | 0.1~10 | <75 (97.7 for $WO_3$) |
| Metal oxide based ECD [55] | >10 | >0.5 | 1~20 | <75 |
| **2D VMT liquid crystalline dispersion (This work)** | >1 | 1.8 | 0.35 | 96.8 |

## Captions for Videos S1-3

**Video S1 | Displayable pixel shown red, green and blue colors.** A screen is used as a backlight, where the color gradually changes from standard red with a RGB color code of (255, 0, 0) to green (0, 255, 0) and to blue (0, 0, 255). An electric field of $4 \times 10^{3}$ V $m^{-1}$ (10 kHz) is applied.

**Video S2 | A displayable billboard controlled by a software.** The array displays uppercase letters T, H and U when the controller clicked the pixel icons shown in the smartphone software.

**Video S3 | A displayable billboard controlled by capturing gestures of the human hand.** The array displays numbers ‘1’, ‘2’ and ‘3’ following the gestures of the controller.

## Supplementary References

1. Kresse G and Furthmuller J. Efficient iterative schemes for ab initio total-energy calculations using a plane-wave basis set. *Phys Rev B* 1996; **54**: 11169.

2. Blochl PE. Projector augmented-wave method. *Phys Rev B* 1994; **50**: 17953.

3. Kresse G and Furthmüller J. Efficiency of ab-initio total energy calculations for metals and semiconductors using a plane-wave basis set. *Comput Mater Sci* 1996; **6**: 15.

4. Perdew JP, Burke K, Ernzerhof M. Generalized gradient approximation made simple. *Phys Rev Lett* 1996; **77**: 3865.

5. Grimme S, Ehrlich S, Goerigk L. Effect of the damping function in dispersion corrected density functional theory. *J Comput Chem* 2011; **32**: 1456.

6. Grimme S, Antony J, Ehrlich S *et al.* A consistent and accurate ab initio parametrization of density functional dispersion correction (DFT-D) for the 94 elements H-Pu. *J Chem Phys* 2010; **132**: 154104.

7. Monkhorst HJ and Pack JD. Special points for brillouin-zone integrations. *Phys Rev B* 1976; **13**: 5188.

8. King-Smith RD and Vanderbilt D. Theory of polarization of crystalline solids. *Phys Rev B* 1993; **47**: 1651.

9. Sheppard D, Xiao P, Chemelewski W *et al.* A generalized solid-state nudged elastic band method. *J Chem Phys* 2012; **136**.

10. O'Konski CT, Yoshioka K, Orttung WH. Electric properties of macromolecules. Iv. Determination of electric and optical parameters from saturation of electric birefringence in solutions. *J Phys Chem* 1959; **63**: 1558.

11. Shen TZ, Hong SH, Song JK. Electro-optical switching of graphene oxide liquid crystals with an extremely large Kerr coefficient. *Nat Mater* 2014; **13**: 394.

12. Peterlin A and Stuart HA. Zur theorie der strmungsdoppelbrechung von kolloiden und groen moleklen in lsung. *Z Phys* 1939; **112**: 1.

13. Peterlin A and Stuart HA. Uber die bestimmung der gre und form, sowie der elektrischen, optischen und magnetischen anisotropie von submikroskopischen teilchen mit hilfe der knstlichen doppelbrechung und der inneren reibung. *Z Phys* 1939; **112**: 129.

14. Taylor EW and Cramer W. Birefringence of protein solutions and biological systems. I. *Biophys J* 1963; **3**: 127.

15. Dozov I, Paineau E, Davidson P *et al.* Electric-field-induced perfect anti-nematic order in isotropic aqueous suspensions of a natural beidellite clay. *J Phys Chem B* 2011; **115**: 7751.

16. Huang Z, Lan T, Dai L *et al.* 2D functional minerals as sustainable materials for magneto-optics. *Adv Mater* 2022; **34**: 2110464.

17. Thurston GB and Bowling DI. The frequency dependence of the Kerr effect for suspensions of rigid particles. *J Colloid Interface Sci* 1969; **30**: 34.

18. Buluy O, Aryasova N, Tereshchenko O *et al.* Optical and X-ray scattering studies of the electric field-induced orientational order in colloidal suspensions of pigment nanorods. *J Mol Liq* 2018; **267**: 286.

19. O'Konski CT. Electric properties of macromolecules. V. Theory of ionic polarization in polyelectrolytes. *J Phys Chem* 1960; **64**: 605.

20. Onsager L. The effects of shape on the interaction of colloidal particles. *Ann NY Acad Sci* 1949; **51**: 627.

21. van der Kooij FM and Lekkerkerker HNW. Formation of nematic liquid crystals in suspensions of hard colloidal platelets. *J Phys Chem B* 1998; **102**: 7829.

22. Ren Y, Wu M, Liu JM. Ultra-high piezoelectric coefficients and strain-sensitive curie temperature in hydrogen-bonded systems. *Natl Sci Rev* 2021; **8**: nwaa203.

23. Li L and Wu M. Binary compound bilayer and multilayer with vertical polarizations: Two-dimensional ferroelectrics, multiferroics, and nanogenerators. *ACS Nano* 2017; **11**: 6382.

24. Wu M and Li J. Sliding ferroelectricity in 2D van der Waals materials: Related physics and future opportunities. *Proc Natl Acad Sci U S A* 2021; **118**: e2200995119.

25. Tuckerman ME and Marx D. Heavy-atom skeleton quantization and proton tunneling in "intermediate-barrier" hydrogen bonds. *Phys Rev Lett* 2001; **86**: 4946.

26. Li XZ, Probert MI, Alavi A *et al.* Quantum nature of the proton in water-hydroxyl overlayers on metal surfaces. *Phys Rev Lett* 2010; **104**: 066102.

27. Nosrati A, Addai-Mensah J, Skinner W. Muscovite clay mineral particle interactions in aqueous media. *Powder Technol* 2012; **219**: 228.

28. Osborn JA. Demagnetizing factors of the general ellipsoid. *Phys Rev* 1945; **67**: 351.

29. Saville DA, Bellini T, Degiorgio V *et al.* An extended Maxwell–Wagner theory for the electric birefringence of charged colloids. *J Chem Phys* 2000; **113**: 6974.

30. Fernández M, Casanova E, Alonso I. Review of display technologies focusing on power consumption. *Sustainability* 2015; **7**: 10854.

31. Li LS and Alivisatos AP. Origin and scaling of the permanent dipole moment in cdse nanorods. *Phys Rev Lett* 2003; **90**: 097402.

32. Dozov I, Goldmann C, Davidson P *et al.* Probing permanent dipoles in cdse nanoplatelets with transient electric birefringence. *Nanoscale* 2020; **12**: 11040.

33. Arenas-Guerrero P, Delgado AV, Donovan KJ *et al.* Determination of the size distribution of non-spherical nanoparticles by electric birefringence-based methods. *Sci Rep* 2018; **8**: 9502.

34. Yin K, Hsiang EL, Zou J *et al.* Advanced liquid crystal devices for augmented reality and virtual reality displays: Principles and applications. *Light Sci Appl* 2022; **11**: 161.

35. Hinds IC, Ridler PJ, Jennings BR. Electric birefringence for monitoring size changes in clay suspensions. *Clay Miner* 1996; **31**: 549.

36. Hristova SH and Zhivkov AM. Electrooptical determination of the isoelectric point of globular proteins: Cytochrome c adsorbed on montmorillonite nanoplates. *Colloids Surf, B* 2019; **176**: 480.

37. Wu ST and Wu CS. High-speed liquid-crystal modulators using transient nematic effect. *J Appl Phys* 1989; **65**: 527.

38. Kumada A, Iwata A, Ozaki K *et al.* Kerr effect in gas and its application to noncontact measurement of electric field. *J Appl Phys* 2002; **92**: 2875.

39. Kamiya T, Matsuoka S, Kumada A *et al.* High voltage measuring apparatus based on kerr effect in gas. *IEEE Trans Dielectr Electr Insul* 2015; **22**: 760.

40. Beevers MS and Khanarian G. The temperature dependence of the Kerr constant of polar liquids. *Aust J Chem* 1980; **33**: 2585.

41. Lee SM and Hauser SM. Kerr constant evaluation of organic liquids and solutions. *Rev Sci Instrum* 1964; **35**: 1679.

42. Schlick MC, Kapernaum N, Neidhardt MM *et al.* Large electro-optic Kerr effect in ionic

liquid crystals: Connecting features of liquid crystals and polyelectrolytes. *ChemPhysChem* 2018; **19**: 2305.

43. Majles Ara MH, Mousavi SH, Mousavi Z *et al.* Investigation of the Kerr effect and third-order susceptibility constants in a nematic liquid crystal. *J Mol Liq* 2011; **161**: 41.

44. Ghanadzadeh A and Beevers MS. The static Kerr effect of two nematic mixtures comprised of pentyl and heptyl cyanobiphenyls in the isotropic phase. *J Mol Liq* 2004; **112**: 141.

45. Hisakado Y, Kikuchi H, Nagamura T *et al.* Large electro-optic Kerr effect in polymer-stabilized liquid-crystalline blue phases. *Adv Mater* 2005; **17**: 96.

46. Haseba Y, Kikuchi H, Nagamura T *et al.* Large electro-optic Kerr effect in nanostructured chiral liquid-crystal composites over a wide temperature range. *Adv Mater* 2005; **17**: 2311.

47. Chen Y, Xu D, Wu ST *et al.* A low voltage and submillisecond-response polymer-stabilized blue phase liquid crystal. *Appl Phys Lett* 2013; **102**: 141116.

48. Pozhidaev EP, Kiselev AD, Srivastava AK *et al.* Orientational Kerr effect and phase modulation of light in deformed-helix ferroelectric liquid crystals with subwavelength pitch. *Phys Rev E* 2013; **87**: 052502.

49. Srivastava AK, Pozhidaev EP, Chigrinov VG *et al.* Vertically aligned ferroelectric liquid crystals with high Kerr constant for field sequential color displays. *J Mol Liq* 2019; **295**: 111054.

50. Pozhidaev EP, Srivastava AK, Kiselev AD *et al.* Enhanced orientational Kerr effect in vertically aligned deformed helix ferroelectric liquid crystals. *Opt Lett* 2014; **39**: 2900.

51. Arenas-Guerrero P, Jiménez ML, Scott K *et al.* Electric birefringence of carbon nanotubes: Single- vs double-walled. *Carbon* 2018; **126**: 77.

52. Kim J, Martinelli L, Lahlil K *et al.* Optimized combination of intrinsic and form birefringence in oriented LaPO4 nanorod assemblies. *Appl Phys Lett* 2014; **105**: 061102.

53. Jimenez ML, Fornasari L, Mantegazza F *et al.* Electric birefringence of dispersions of platelets. *Langmuir* 2012; **28**: 251.

54. Cai G, Wang J, Lee PS. Next-generation multifunctional electrochromic devices. *Acc Chem Res* 2016; **49**: 1469.

55. Ke YJ, Chen JW, Lin CJ *et al*. Smart windows: Electro-, thermo-, mechano-, photochromics, and beyond. *Adv Energy Mater* 2019; **9**: 1902066.

56. Bisoyi HK and Li Q. Light-driven liquid crystalline materials: From photo-induced phase transitions and property modulations to applications. *Chem Rev* 2016; **116**: 15089.

57. Wen C-H, Gauza S, Wu S-T. Ultraviolet stability of liquid crystals containing cyano and isothiocyanato terminal groups. *Liq Cryst* 2010; **31**: 1479.

58. Wu W, Wang M, Ma J *et al.* Electrochromic metal oxides: Recent progress and prospect. *Adv Electron Mater* 2018; **4**: 1800185.

59. Chen HW, Lee JH, Lin BY *et al.* Liquid crystal display and organic light-emitting diode display: Present status and future perspectives. *Light Sci Appl* 2018; **7**: 17168.
